# Long-term growth comparison studies of FBS and FBS alternatives in six head and neck cell lines

**DOI:** 10.1371/journal.pone.0178960

**Published:** 2017-06-07

**Authors:** Chih-Yeu Fang, Chung-Chun Wu, Chia-Lang Fang, Wei-Yu Chen, Chi-Long Chen

**Affiliations:** 1 Department of Pathology, Wan Fang Hospital, Taipei Medical University, Taipei, Taiwan; 2 Department of Pathology, School of Medicine, College of Medicine, Taipei Medical University, Taipei, Taiwan; 3 National Institute of Cancer Research, National Health Research Institutes, Miaoli, Taiwan; 4 Department of Pathology, Taipei Medical University Hospital, Taipei Medical University, Taipei, Taiwan; Medical University of South Carolina, UNITED STATES

## Abstract

Fetal bovine serum (FBS) is depended upon by investigators as an indispensable supplement in cell and tissue culture systems. Due to increased demand and limited availability, the price of FBS has increased by greater than 300% in the past few years. In addition, there are ethical and scientific controversies about the collection and use of FBS in culture systems. In response to the shortage of FBS, many FBS alternative serum products have been developed. Although many have claimed comparable performance to FBS, their support of long-term cell growth and effects on cell phenotype have not been revealed. In this study, we examined the performances of six bovine calf serum-based FBS alternatives in six head and neck cell lines and compared them with FBS. The results indicate that some of these sera had growth promoting capabilities comparable or superior to that of FBS. Additionally, these alternative sera supported long-term (30 passages) growth of tested cells and exhibited plating efficiencies comparable to that of FBS. Cells cultured in alternative sera also exhibited comparable anchorage-independent growth and similar drug inhibition responses in FBS. Still, caution should be taken in choosing suitable sera given that changes in cell morphology and variations in chemotactic responses were noted for cells maintained in certain sera. These FBS alternatives are more readily available, cost less, and are associated with less ethical concerns, thus making them attractive alternatives to FBS in cell culture systems.

## Introduction

Cell and tissue culture is an indispensable methodology for the research community and biotechnology, pharmaceutical and diagnostics industries. The use of cell and tissue culture has been increasing exponentially since its introduction. In most cases, the supplementation of basal culture media with animal-derived products, mainly animal serum, is essential in cell culture for proper cell growth. The major functions of serum in culture media include providing hormone factors for cell growth and proliferation; promoting cell differentiation; supplying transport proteins, essential nutrients, trace elements, adherence and extension factors; and stabilizing and detoxifying factors needed for maintaining a favorable growth environment [1]. Among several common animal sera, fetal bovine serum (FBS) has been the most used cell culture supplement. FBS has very low level of antibodies and contains more growth factors than calf and adult bovine serum, thus allowing the propagation of most types of human and animal cells. Although a number of synthetic serum-free media have been developed, FBS continues to be depended upon by numerous investigators given that it works well in cell culture systems.

FBS is a by-product of cattle husbandry. FBS is obtained from blood drawn from a bovine fetus when a pregnant cow is processed at the slaughterhouse. This procedure had raised ethical concerns due to the potential suffering of the fetus by the collection practice [2]. In addition, there are several scientific controversies about using animal serum in culture systems. FBS is an ill-defined mixture of components that contains thousands of constituents and can contain contaminants, such as endotoxins, mycoplasma, viruses or prion proteins. Due to geographical and seasonal variations, serum could vary significantly from lot to lot, and consistency among the components is not maintained. FBS from different source areas could have as much as 1-fold discrepancy in growth promotion of certain cell types [3]. Despite ethical and scientific concerns, the unsteady supply of FBS is itself a major problem. Environmental factors, such as drought, along with governmental farm policies, beef and dairy prices, feed costs, and the outbreak of diseases, all contribute to fluctuations in the FBS supply [4, 5]. In recent years, the demand for FBS in China, India, South Korea, and Middle East countries has increased significantly [6]. Due to the limitation of its availability, the price of FBS has increased by greater than 300% in the past few years [6].

Given the current shortage and high price of FBS, some researchers are searching for other options for cell and tissue culture. The demand for alternatives to FBS has led to the development of several commercial products that claimed to have similar or superior performance to FBS. A major portion of these “FBS alternatives” is bovine calf serum-based products supplemented with chemically defined components, including vitamins, amino acids, trace metals, and other small molecules that stimulate cell growth and proliferation. The use of serum products derived from mature cattle as a replacement may mitigate the ethical concerns posed by the use of FBS [7]. In addition, these products are more readily available, cost less, and exhibit good uniformity between lots [3].

The development of FBS alternatives began early in the 1990s. Although studies have demonstrated that several products support the growth of cells similar to that of FBS [8, 9], their use was not widely popularized. Most cell culture scientists still use FBS despite the shortage and high price. Although the business associated with FBS is large and worldwide, the market is only loosely regulated [10, 11]. Recently, a case of FBS supply fraud was reported and is considered to represent the tip of the iceberg [12]. This event should prompt investigators to rethink whether they should rely on FBS as the only choice or whether other options are available that might work as well as FBS in culture systems. Given its reduced cost and increased availability, calf serum-based FBS alternatives are worthy of investigation as substitutions for FBS. Additionally, given that most cell lines were constructed and/or maintained in FBS-containing medium, the switch to calf serum-based alternatives might have less impact on cells compared with serum products based on other animals or serum-free products. The most common concern about FBS alternatives is the lack of solid evidence indicating that these sera exhibit equal performance as FBS. Although the manufacturers provided documents claiming that these FBS replacements achieved results similar to FBS in the culture of certain cell lines [3, 13], the number of generations these cells had been cultured or whether these sera support long-term cell growth is not known. In addition to growth assays, data indicating that the phenotype of cells in alternative sera is the same as in FBS are not available, which is a major concern for many potential users. Here, we studied the growth-promoting capability of six bovine calf serum-based alternatives and compared their performance with FBS in five head and neck squamous carcinoma cells and one dysplastic oral keratinocyte cell lines. These cells were cultured in FBS alternatives for a total of 30 serial passages to assess their ability to support long-term growth. The morphology of cells cultured in FBS and alternative sera was also assessed. A plating efficiency assay was conducted to determine the ability of alternative sera to support the growth of cells under low cell density conditions. Finally, several functional assays were performed to reveal the fundamental differences between FBS and serum alternatives. Our results demonstrated that some of these FBS alternatives could support long-term growth of tested cells without discernible morphological changes. These alternative sera performed similar or even superior to FBS in certain cells, thus making them an attractive option for replacing FBS in cell and tissue culture.

## Materials and methods

### Cell lines, culture media and sera

NPC-TW01 (TW01) and HONE-1 are nasopharyngeal carcinoma cell lines [14, 15]. OECM-1 is a gingival squamous carcinoma cell line [16]. FaDu is a hypopharyngeal carcinoma cell line [17]. SCC25 is a tongue squamous carcinoma cell line [18]. DOK is a non-tumorigenic dysplastic oral keratinocyte cell line [19]. FaDu and SCC25 were obtained from the Bioresource Collection and Research Center, Taiwan. The DOK cell line was obtained from the European Collection of Authenticated Cell Cultures, UK. TW01, HONE-1 and OECM-1 were cultured in Dulbecco's Modified Eagle's medium (DMEM) supplemented with 10% serum. DOK was cultured in DMEM with 5 μg/ml hydrocortisone and 10% serum. FaDu was cultured in Eagle's minimum essential medium supplemented with non-essential amino acids and 10% serum. SCC25 was cultured in 1:1 mixture of DMEM and Ham's F12 medium and supplemented with 400 ng/ml hydrocortisone and 10% serum. The sera used in this study included fetal bovine serum (FBS, Gibco 26140, lot 1566368, Thermo Fisher, USA), newborn calf serum (NBCS, Gibco 16010, lot 1517932, Thermo Fisher, USA, and HyClone SH30118, lot ABA212935, GE Healthcare, USA), bovine calf serum (CS, Gibco 16170, lot 1517948, Thermo Fisher, USA), and iron-supplemented calf serum (ICS, Gibco 10317, lot 1645933, Thermo Fisher, USA). Three bovine serum-based alternatives were used as serum substitutes in this study: Fetalgro bovine growth serum (FG, RMBIO FGR-BBT, lot 20150302FG, Rocky Mountain Biologicals, USA), Cosmic calf serum (CCS, HyClone SH30087, lot AZD189128, GE Healthcare, USA), and FetalClone III serum (FC3, HyClone SH30109, lot AAM211834, GE Healthcare, USA). All sera were aliquoted and added into the growth medium without heat-inactivation or any additional treatment. The FBS used in this study (Gibco 26140, lot 1566368, Thermo Fisher, USA) promotes vigorous growth of TW01 and HONE-1 cells as demonstrated in a prior screening of three batches of FBS from three vendors (data not shown).

### Brief adaptation of cells into test sera

To minimize the impact of the serum switch for the cultured cell lines, five passages of sequential serum adaptation was performed. Cells originally cultured in 10% FBS-containing medium were subcultured in medium containing 8% FBS and 2% destination serum. After 3 days (as cell reached near confluence), cells were then subcultured in medium with 6% FBS and 4% destination serum followed by 4% FBS and 6% destination serum, 2% FBS and 8% destination serum, and finally medium containing 10% destination serum. The first passage of cells in 10% destination serum-containing medium is designated P1 (passage 1) cells.

### Long-term cell proliferation assay

The cells in the test serum medium were cultured in triplicate (three individual wells) with 1.5x10^5^ cells in 3 ml medium per well of a 6-well plate. After 3 days of growth, cells were trypsinized, counted, and subcultured into new wells. A total of 30 serial passages of this 3-day interval subculture were performed for the six cell lines, each in six sera. Total cell number in each well after 3 days of growth was determined using the automated cell counter Countess (Invitrogen, Thermo Fisher, USA). For cell morphology analysis, subcultured cells at 48 h were photographed under a microscope.

### Low-density plating efficiency assay

To determine the plating efficiency of each serum under low-cell density conditions, a modified procedure was followed as described previously [20]. Cells were trypsinized, counted, and diluted to plate 500 cells per well of a 12-well plate in 2 ml of growth medium containing the test serum at a concentration of 10%. After 4 days, another 1 ml of the same medium was added. On the 8th day, the cultured cells were rinsed with PBS and fixed with cold methanol. Colonies with greater than 64 cells were counted under a microscope. Plating efficiency was analyzed in passage 25 (P25) cells.

### Cell migration assay

Cell migration assays were performed using Oris Migration Assay kits (Platypus Technologies, USA) as described previously [21]. Briefly, 5 × 10^4^ cells were inoculated in each well of a 96-well plate in the presence of the stoppers. The central stopper was removed after 12 h of incubation to allow the cells to migrate into the central area. Photographs were obtained to document the unoccupied area at t = 0 h (as stopper removed). The cells that had migrated into the central area were then photographed at 32 h for TW01 and HONE-1 cells and at 48 h for FaDu cells. The area of the unoccupied central region of each well was determined by ImageJ (https://imagej.nih.gov/ij/), and the percentage of migration was calculated using the equation: % Migration = [(Area_t = 0_ — Area_t = Δ_) × Area_t = 0_] × 100%, where Δ = 32 or 48 h [22].

### Cell invasion assay

Invasion assays were performed using 24-well culture inserts with 8.0-μm pores (BD Biosciences, USA) as described previously [23]. Briefly, the transwell membranes were coated with Matrigel (Corning Life Sciences, USA). Then, 1 × 10^5^ cells were seeded onto the Matrigel-coated membranes, and the inserts were incubated in 24-well plates. Cells in the inserts were cultured in medium containing 2% serum, whereas the lower wells were filled with medium containing 10% serum as an attractant. After 48-h incubation, the membranes were fixed with methanol and stained with 0.05% crystal violet. Cells on the upper surface of the membrane were removed using a cotton swab. Cells that had invaded and transmigrated to the lower surface of the polycarbonate membrane were photographed under an inverted microscope, and the cell number was calculated as described [23].

### Anchorage-independent colony formation assay

The anchorage-independent growth of cells in alternative sera was estimated by a soft-agar colony formation assay [24]. Single-cell suspensions of 3 × 10^3^ cells were plated per well of a 12-well plate in 1 ml of medium containing 10% tested serum and 0.36% agar on a layer of 0.5 ml of the same medium containing 0.75% agar. Three weeks after plating, the colonies were counted under a microscope.

### Cell cytotoxicity assay

Cell viability was determined using the standard MTT assay. Briefly, cells were seeded in 96-well plates at a density of 2 × 10^4^ cells/100 μl per well overnight and then treated with cisplatin (*cis*-Diamineplatinum (II) dichloride; Sigma-Aldrich, USA) at various concentrations for 72 h. At the end of the treatment, 20 μl of 3-(4,5-dimethylthiazol-2-yl)-2,5-diphenyltetrazolium bromide (MTT; 5 mg/ml; Sigma-Aldrich) was added to the culture medium, and the incubation continued at 37°C for 2 h. After incubation, the supernatant was removed. Dark formazan formed was dissolved in DMSO. Then, formazan was measured using a microplate reader at an absorption wavelength of 540 nm.

### Expression profiling of transcription factor by RT^2^-PCR Array analysis

cDNA was synthesized from 0.5 μg RNA using the RT^2^ First Strand kit, according to the manufacturer’s instructions (Qiagen, Germany). Human Transcription Factors RT^2^ Profiler PCR Arrays (PAHS-075Z, Qiagen) were used to analyze the differential expression of transcription factors. Briefly, cDNA was added to the RT^2^ SYBR Green qPCR Master Mix and aliquoted onto each well of the 96-well RT^2^ Profiler PCR Array plate. A Roche LightCycler 480 instrument (Roche Molecular Systems, USA) was used for mRNA quantification per the conditions suggested by the RT^2^-PCR manual. Analysis of the PCR array data was performed using an online data analysis program provided by the manufacturer (http://www.qiagen.com/shop/genes-and-pathways/data-analysis-center-overview-page/). The sample data of each array were normalized to housekeeping genes as suggested in the RT^2^ instruction manual. The transcript level of each candidate gene was quantified according to the ΔΔCt method. Ct values > 35 were not included in the analysis and considered as negative.

### Western blot analysis

Cell lysates were prepared by lysis of monolayer cells in RIPA buffer. Lysates were separated in a 10% polyacrylamide gel and transferred onto a PVDF membrane. The blot was then probed with primary and secondary antibodies using a standard procedure, as described previously [25]. The expression profile of the proteins was visualized using a Western Lightening-ECL kit (PerkinElmer, USA). Antibodies against ELK1, c-Fos, SMAD4, (Genetex, USA), β-catenin/CTNNB1, and GAPDH (Cell Signaling, USA) were used as the primary antibodies in these analyses.

### Statistical analysis

Differences between multiple groups were analyzed by one-way ANOVA using Dunnett’s method for pairwise comparisons. *p*<0.05 was considered statistically significant.

## Results

### Newborn calf serum did not provide supportive conditions for the growth of head and neck cell lines

**Fig 1:**
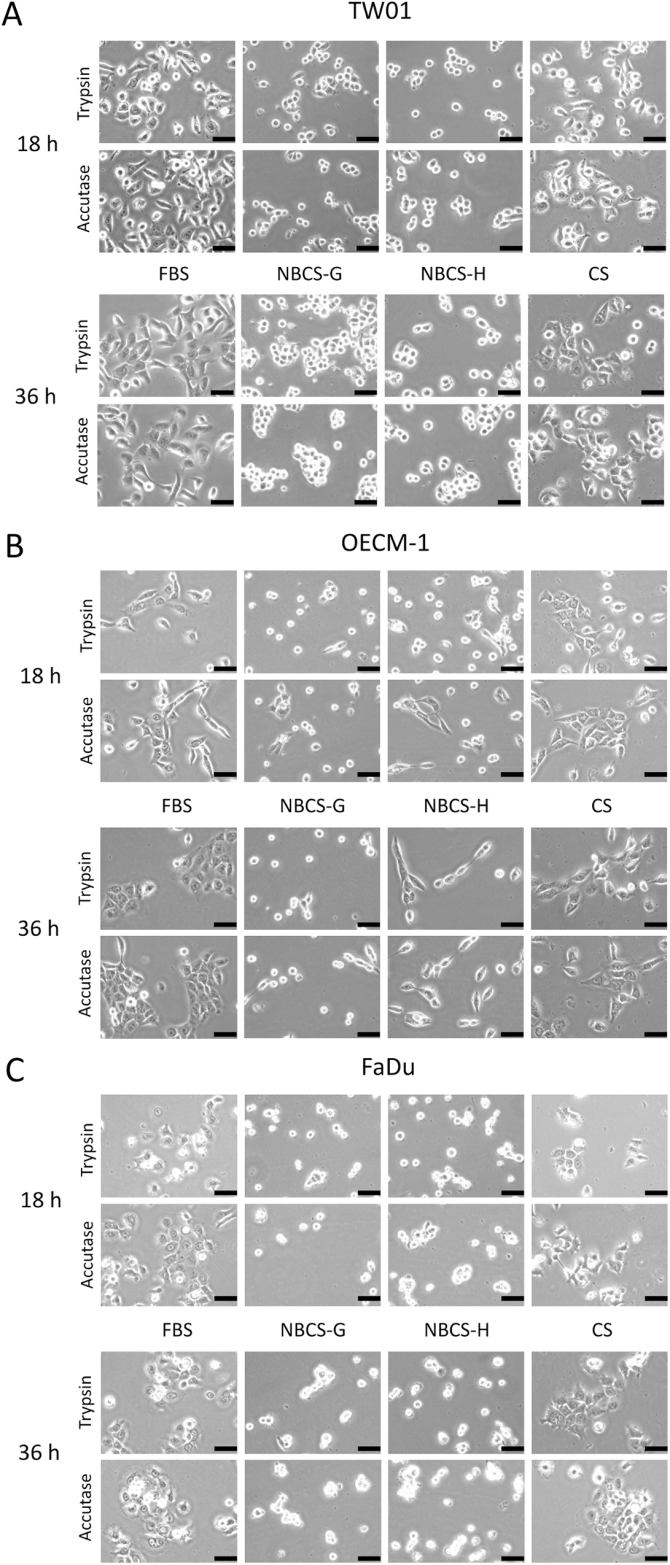
Morphology of cells cultured in FBS, NBCS, and CS. Cells were detached with trypsin (0.05%)-EDTA or Accutase and cultured in medium containing fetal bovine serum (FBS), newborn calf serum (NBCS), and bovine calf serum (CS). Cultured cells were photographed at 18 and 36 h after seeding. (A) TW01, (B) OECM-1, and (C) FaDu cells. Black bar represents 50 μm. NBCS-G: Gibco NBCS; NBCS-H: HyClone NBCS.

**Fig 2:**
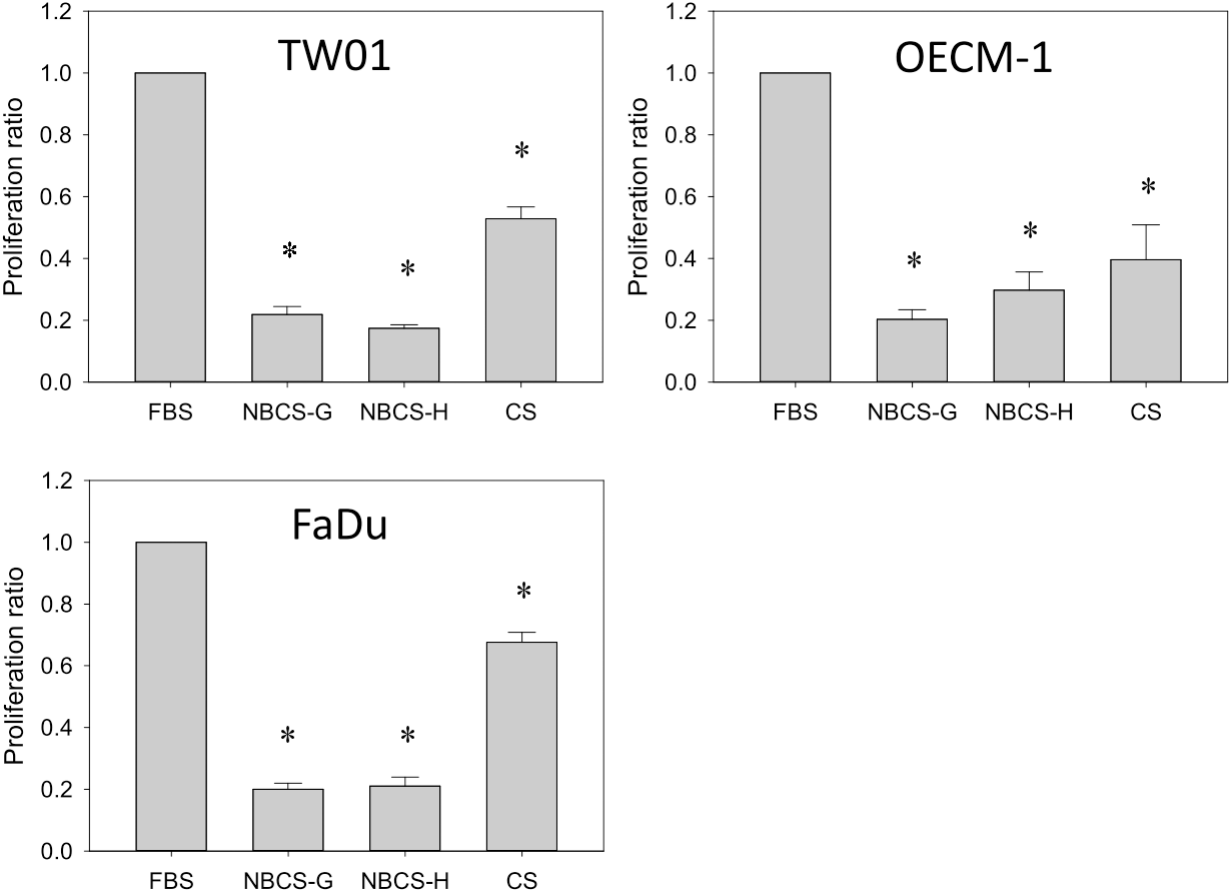
Newborn calf serum did not provide favorable conditions for the growth of head and neck cell lines. The proliferation of cells cultured in NBCS and CS at 72 h was presented as the relative proliferation ratio of cells in FBS (1.0-fold). Data indicate the average value of triplicates (mean ± SD). *:p<0.001 compared with cells cultured in FBS. NBCS-G: Gibco NBCS; NBCS-H: HyClone NBCS.

Newborn calf serum (NBCS) is collected from calves typically 14 days old or younger. NBCS is intrinsically more close to FBS than calf and adult bovine serum, which are collected from cattle less than 12 months and greater than 12 months old, respectively [26]. NBCS is suggested by many serum vendors as a cost-effective replacement for FBS. Here, our study demonstrated that NBCS-containing medium may not provide favorable conditions for supporting the proliferation of head and neck cancer cell lines. Compared with FBS and calf serum (CS), cells cultured in NBCS medium revealed delayed attachment to the culture surface (Fig 1). Although the cells did attach to the plate after longer incubation times (36 h), they exhibited a constrained morphology and did not extend as well as cells cultured in the FBS and CS. A previous study noted that certain sera may have low trypsin inhibitor activity and may affect cell attachment if the trypsin used to detach cells is not properly inactivated [8]. Thus, a duplicated study was performed by using Accutase (Mediatech, Corning Life Sciences, USA) as the detachment reagent. Accutase promotes gentle detachment that does not require neutralization and has demonstrated high cell viability even in serum-free cultures. However, similar results were observed in TW01, OECM-1 and FaDu cells regardless of detach treatments (Fig 1). Despite gentle treatment by Accutase, cells cultured in NBCS-containing medium still exhibited delayed attachment and poor extension. This result indicated that trypsin is unlikely the factor that contributed to the poor cell attachment/extension in NBCS medium. Additionally, to avoid the variation of a certain lot of NBCS that might affect the result of this study, NBCS from two different vendors (Gibco, NBCS-G, and HyClone, NBCS-H) of different sources (Gibco-New Zealand; HyClone-United States) were included. However, the cells still exhibited delayed attachment, low extension, and low proliferation in both NBCS-containing media (Fig 1). After 3 days of culture, the cell number in NBCS was even lower than that of CS (Fig 2). Among three tested cells, FaDu eventually attached to the surface and started to proliferate but at an exceptionally slow rate. FaDu cells achieved 50% confluence in NBCS after 7–8 days, whereas these cells achieved 100% confluence in 3 days in FBS (data not shown). These results indicate that NBCS may not be a good supplement to culture head and neck cell lines. Thus, newborn calf serum was not included in the following studies.

### Long-term proliferation and cell morphology analysis of cells in alternative sera

Numerous manufacturers of FBS alternatives provide documents claiming that their serum replacements perform as well as FBS in cell culture [3, 13]. However, the number of generations that the cells had been cultured for and whether the different sera support long-term cell growth are unknown. Cells switched to serum other than FBS might exhibit similar replication rates during the first few passages; however, a significant increase in doubling time after 8 passages was reported [27]. Therefore, in this study, we tested five FBS replacement candidates, including bovine calf serum (CS), iron-supplemented bovine calf serum (ICS), FetalClone III (FC3), Cosmic calf serum (CCS), and Fetalgro (FG), in six head and neck cell lines and compared their growth-promoting capabilities with FBS in a 30 serial-passage culture. FC3, CCS, and FG are bovine calf serum-based alternative supplements with proprietary growth-promoting factors. The morphological characteristics of cells cultured in these sera were also analyzed.

#### NPC-TW01

**Fig 3:**
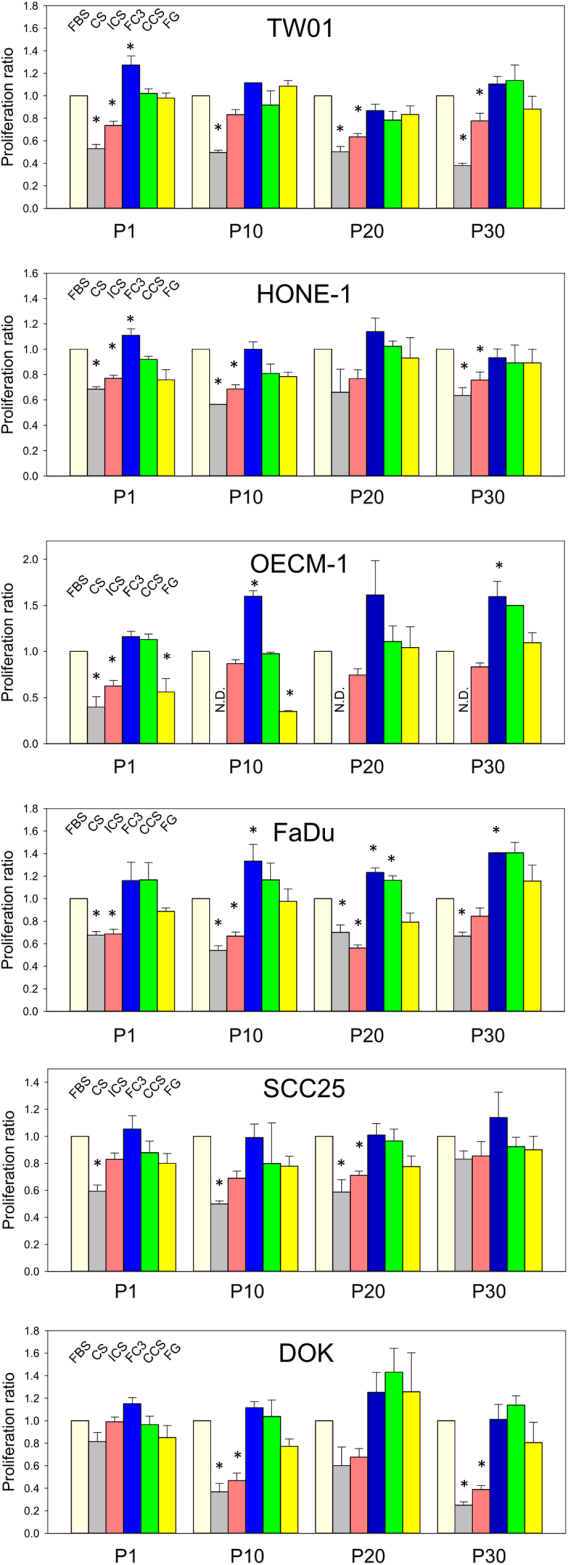
Long-term proliferation comparison of cells in FBS and alternative sera. The proliferation profiles of cells cultured in fetal bovine serum (FBS), calf serum (CS), iron-supplemented calf serum (ICS), FetalClone III (FC3), Cosmic calf serum (CCS), and Fetalgro (FG) were presented at passages 1, 10, 20, and 30. Cells cultured in FBS were adjusted as the baseline (1.0-fold), and the relative proliferation ratio of cells in other sera was determined accordingly. Data indicate the average value of triplicates (mean ± SD). *:p<0.01 compared with cells cultured in FBS at the same passage. N.D.: not determined for OECM-1 cells cultured in CS due to subculture termination at passage 10.

**Fig 4:**
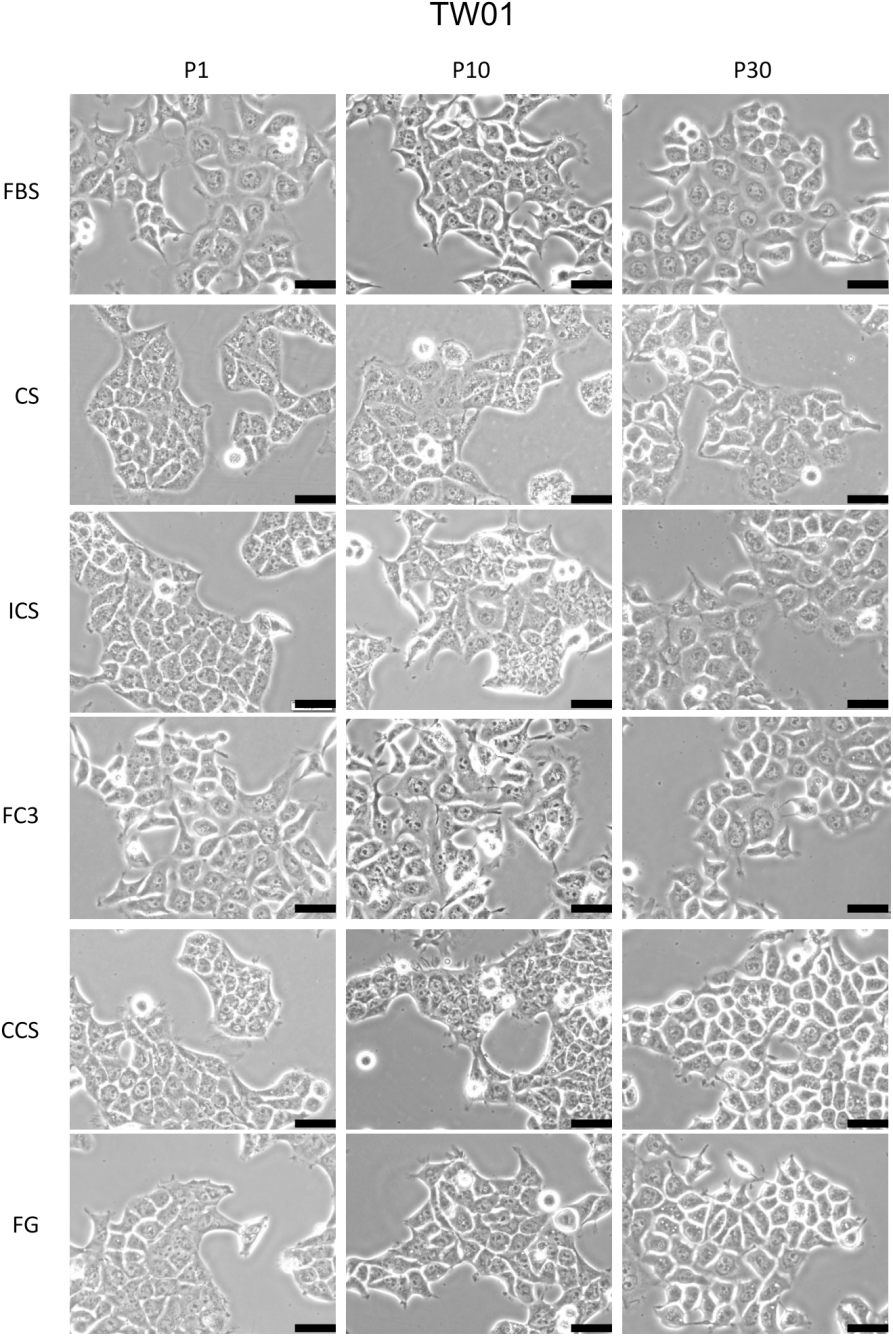
Morphology analysis of TW01 cells cultured in alternative sera. The morphology of cells cultured in fetal bovine serum (FBS), calf serum (CS), iron-supplemented calf serum (ICS), FetalClone III (FC3), Cosmic calf serum (CCS), and Fetalgro (FG) were presented at passages 1, 10, and 30. Pictures were obtained at 200× magnification. Black bar represents 50 μm.

NPC-TW01 (TW01) is a nasopharyngeal carcinoma cell line [14]. Although fluctuations were noted, TW01 cells cultured in FC3, CCS, and FG exhibited growth rates comparable to that of FBS throughout the 30 passages (Fig 3, TW01). In contrast, cells in CS and ICS had constant lower proliferation profiles compared with FBS. ICS exhibited approximately 80% potency, whereas CS was only 50% effective as FBS in TW01 cell culture. All five sera supported TW01 growth throughout the 30 passages of subculture. However, in terms of cell morphology, cells cultured in CS revealed significant increases in cytosolic vacuoles and granularity after P1 (Fig 4). Cells cultured in ICS also exhibited increased granularity in the cytosol, but this phenomenon was not apparent at P30. Cells cultured in CS and ICS appeared more “tame” (less stretched lamellipodia) and were more compact compared with cells grown in FBS. For FC3, no discernible cell morphology change was noted; the cells appeared similar to those cultured in FBS. TW01 cultured in CCS and FG exhibited a slight increase in granularity in the cytosol, and the cell colonies were compact in contrast to those grown in FBS (Fig 4).

#### HONE-1

**Fig 5:**
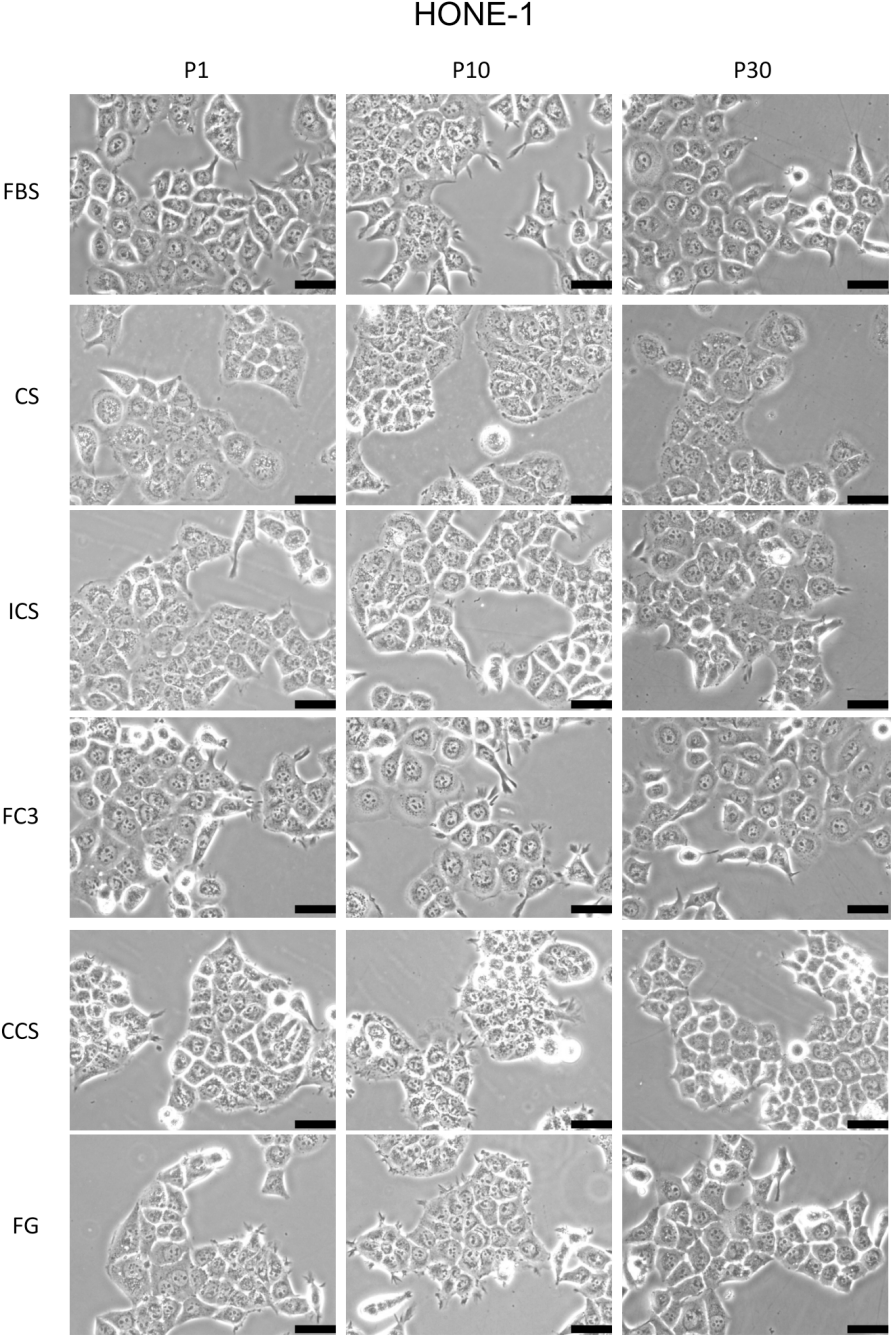
Morphology analysis of HONE-1 cells cultured in alternative sera. The morphology of cells cultured in fetal bovine serum (FBS), calf serum (CS), iron-supplemented calf serum (ICS), FetalClone III (FC3), Cosmic calf serum (CCS), and Fetalgro (FG) were presented at passages 1, 10, and 30. Pictures were obtained at 200× magnification. Black bar represents 50 μm.

HONE-1 is a nasopharyngeal carcinoma cell line [15]. Cells cultured in FC3 exhibited equal or somewhat better performance than FBS (Fig 3, HONE-1). Cells grown in CCS and FG exhibited slightly reduced performance (80–90%) compared with those grown in FBS in the initial ten passages but achieved similar performance to those grown in FBS in the P20 and P30 tests. Although the cells had briefly adapted to the sera (see methods section), it seems that increased passage times may further enhance the adaptation of these cells in the replacement serum. Cells cultured in CS and ICS exhibited reduced proliferation (approximately 70% and 80%, respectively) compared with FBS. All sera were able to support the growth of HONE-1 throughout the 30 passages. Similar to TW01 cells, no discernible cell morphology changes in HONE-1 cells cultured in FC3 were noted compared with FBS (Fig 5). Cells cultured in CS and ICS exhibited increased granularity, and CCS and FG cell colonies appeared more compact compared with cell colonies grown in FBS.

#### OECM-1

**Fig 6:**
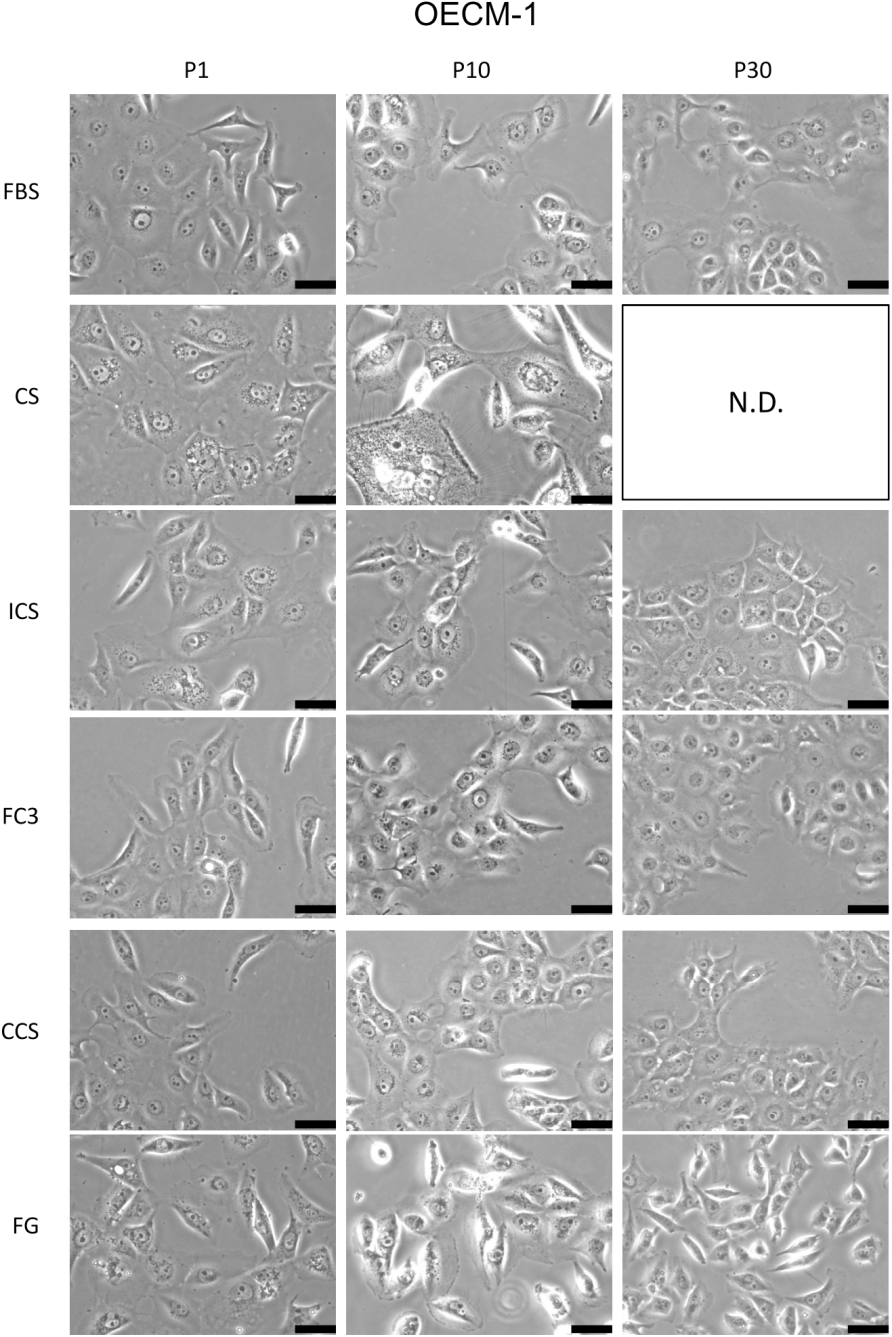
Morphology analysis of OECM-1 cells cultured in alternative sera. The morphology of cells cultured in fetal bovine serum (FBS), calf serum (CS), iron-supplemented calf serum (ICS), FetalClone III (FC3), Cosmic calf serum (CCS), and Fetalgro (FG) were presented at passages 1, 10, and 30. Pictures were obtained at 200× magnification. Black bar represents 50 μm. N.D.: not determined for OECM-1 cells cultured in CS due to subculture termination at passage 10.

OECM-1 is a gingival squamous carcinoma cell line. Except at P1, the growth rate of cells cultured in FC3 surpassed (>150%) FBS at P10, P20, and P30 (Fig 3, OECM-1), and no discernible cell morphology change was observed compared with FBS (Fig 6). Cells grown in CCS had a growth rate identical to FBS at P1, P10, and P20 and outperformed FBS at P30. There was also no noticeable change in the morphology of OECM-1 in CCS compared with FBS. A trend of decreased proliferation was noted in OECM-1 cells cultured in FG from P1 to P10, but the cell number was restored and comparable to that of FBS at P20 and P30. However, a remarkable morphological change was noted. P1 cells cultured in FG exhibited increased cytosolic vacuoles and granularity (Fig 6). At P10, the cells appeared enlarged, elongated, and deformed. At P30, when the cell number in FG was restored, there was a large portion of spindle-shape cells, and the cells were loosely dispersed instead of forming cell-cell contact colonies as observed in FBS. The decrease and subsequent restoration of the deformed OECM-1 cells may indicate that the long-term passage of cells in FG “selected” a specific subset of OECM-1 cells that adapted to the FG serum with striking phenotypic changes. OECM-1 cells cultured in CS had a very low proliferation rate. P1 cells exhibited increased vacuoles and granularity, whereas the P10 cells were massively enlarged, granulated, and degenerated. Their growth was too slow to warrant additional subcultures. Thus, the proliferation assay of OECM-1 in CS was terminated at P10. Intriguingly, cells cultured in ICS had a much better profile compared with cells in CS. Although initially lower, the performance of ICS was approximately 80% that of FBS, and the cells only exhibited an increase in granularity in the cytosol without apparent cell deformation during the 30-passage subculture.

#### FaDu

**Fig 7:**
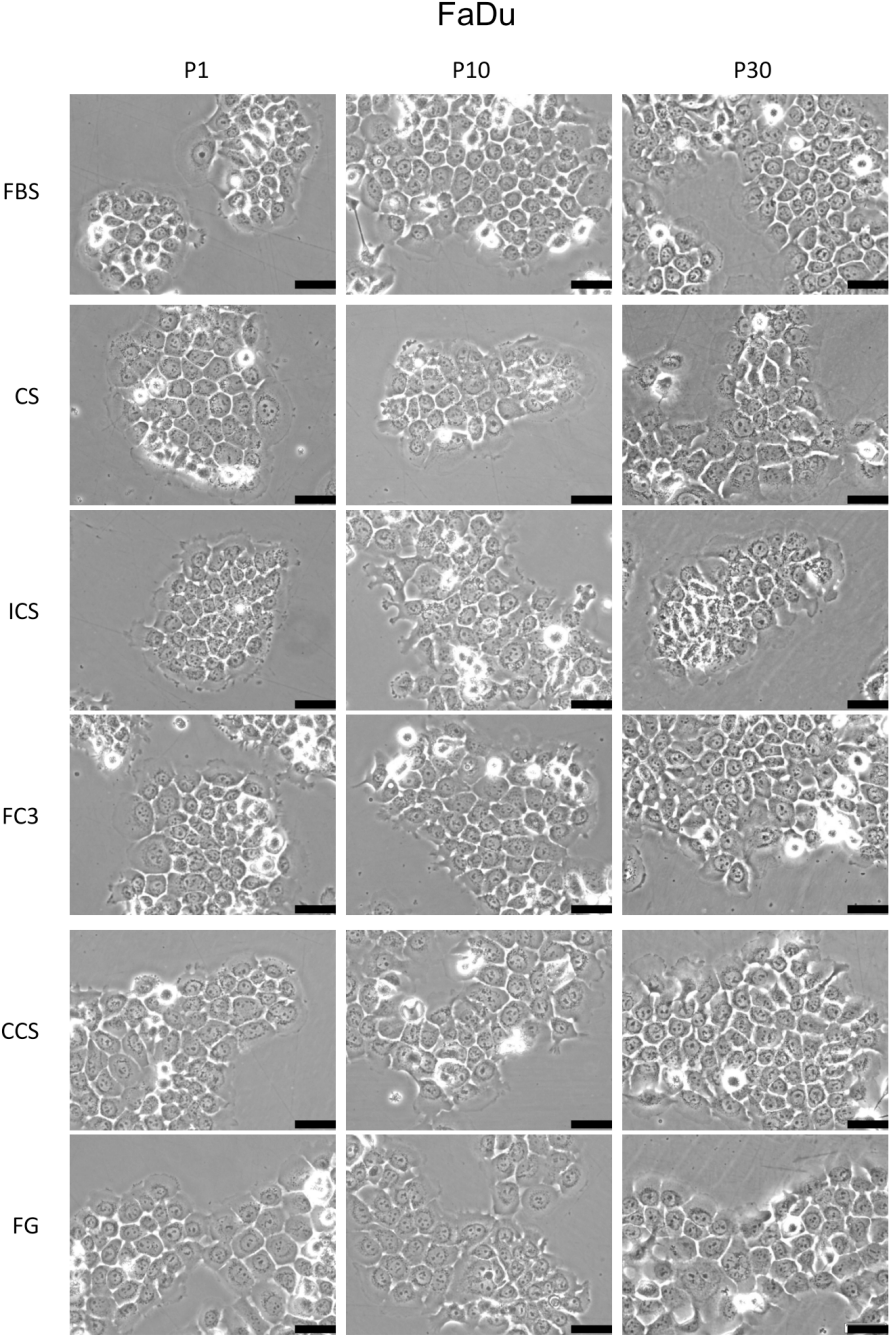
Morphology analysis of FaDu cells cultured in alternative sera. The morphology of cells cultured in fetal bovine serum (FBS), calf serum (CS), iron-supplemented calf serum (ICS), FetalClone III (FC3), Cosmic calf serum (CCS), and Fetalgro (FG) were presented at passages 1, 10, and 30. Pictures were obtained at 200× magnification. Black bar represents 50 μm.

FaDu is a hypopharyngeal carcinoma cell line [17]. Cells cultured in FC3 and CCS exhibited a slightly superior performance (120–140%) compared with FBS (Fig 3, FaDu). Cells maintained in FG had a growth rate similar to that of FBS. The performance of CS and ICS was consistently lower (70–80%) than FBS. All sera supported the growth of FaDu cells throughout the 30 passages without significant changes in cell morphology (Fig 7). Only a minor increase in intracellular granularity was noted in P30 cells cultured in CS. FaDu appears to be a robust cell line that proliferated well in all tested sera (except newborn calf serum, Fig 2).

#### SCC25

**Fig 8:**
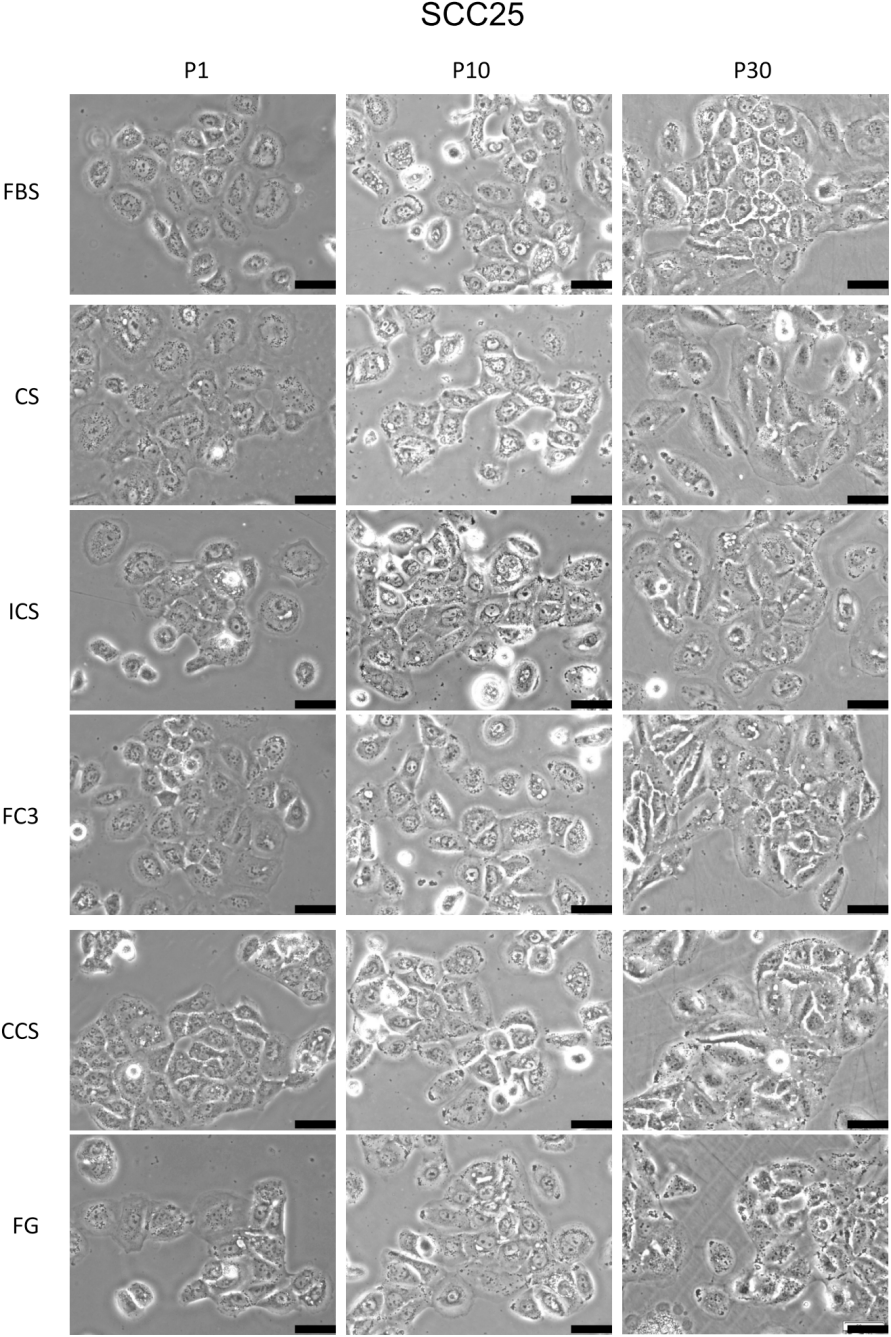
Morphology analysis of SCC25 cells cultured in alternative sera. The morphology of cells cultured in fetal bovine serum (FBS), calf serum (CS), iron-supplemented calf serum (ICS), FetalClone III (FC3), Cosmic calf serum (CCS), and Fetalgro (FG) were presented at passages 1, 10, and 30. Pictures were obtained at 200× magnification. Black bar represents 50 μm.

SCC25 is a tongue squamous carcinoma cell line [18]. Cells cultured in FC3 and CCS had generally identical performance to FBS (Fig 3, SCC25). The proliferation ratio of cells grown in FG was slightly reduced (80–90%) compared with FBS. Cells maintained in CS and ICS had low performance (<80%) at initial passages but the performance was improved to near 90% of FBS at P30. SCC25 intrinsically exhibit multiple granules in the cytosol. No significant morphological change was observed in SCC25 cells cultured in all five sera during the 30-passage subculture (Fig 8).

#### DOK

**Fig 9:**
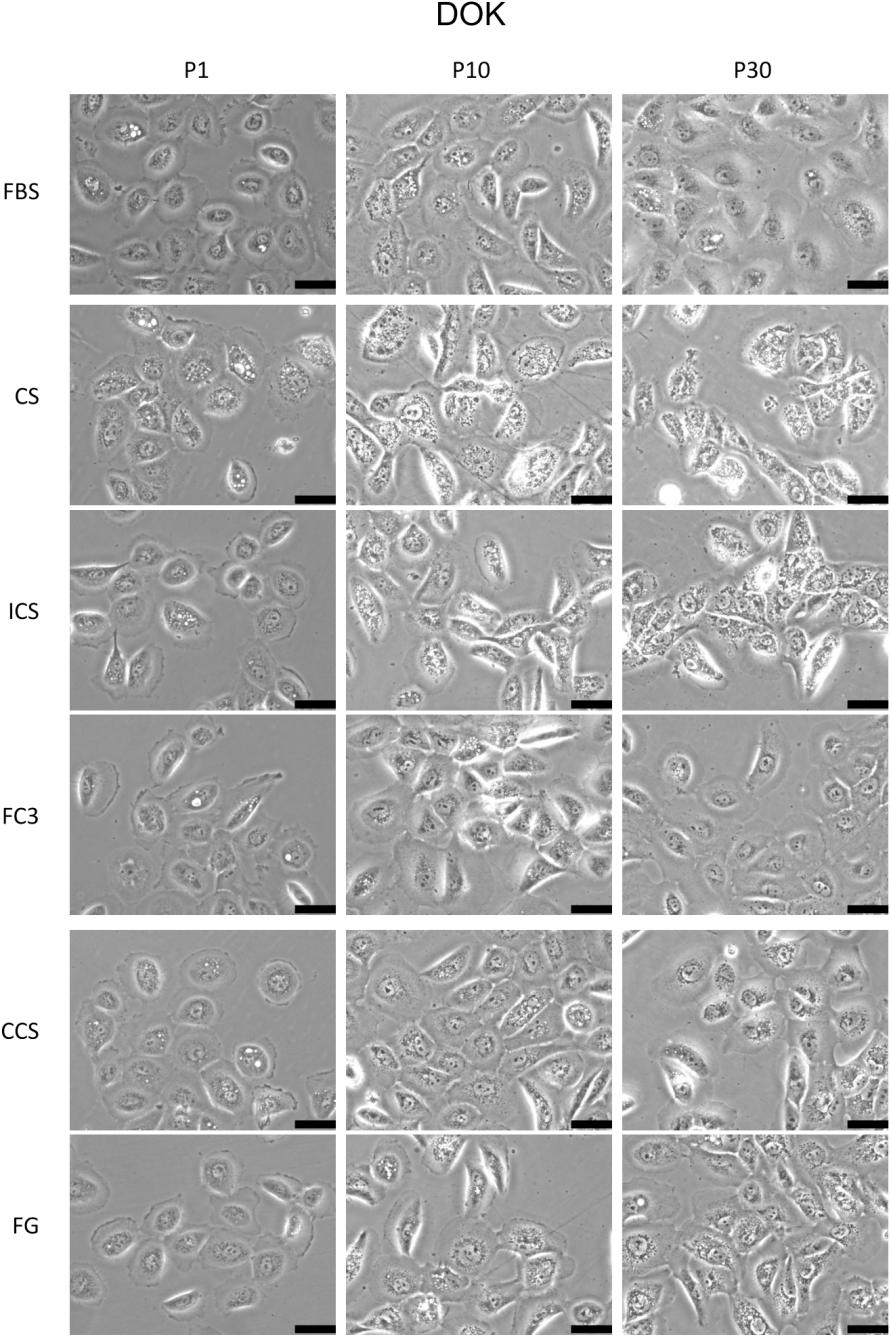
Morphology analysis of DOK cells cultured in alternative sera. The morphology of cells cultured in fetal bovine serum (FBS), calf serum (CS), iron-supplemented calf serum (ICS), FetalClone III (FC3), Cosmic calf serum (CCS), and Fetalgro (FG) were presented at passages 1, 10, and 30. Pictures were obtained at 200× magnification. Black bar represents 50 μm.

DOK is a dysplastic oral keratinocyte cell line [19]. This cell line is not tumorigenic in nude mice and requires high concentrations of hydrocortisone (5 μg/ml) in medium to maintain proper cell proliferation. The DOK proliferation rate is also reduced compared with other carcinoma cell lines tested in this study (data not shown). Cells cultured in FC3 and CCS exhibited comparable performance to FBS; however, an increased rate was observed at P20 (Fig 3, DOK). The proliferation ratio of DOK in FG is slightly less (80%) than FBS. Cells cultured in CS and ICS performed similar to cells grown in FBS at initial passages but declined significantly at P10, P20, and P30. No significant morphological changes were observed in DOK cells in FC3 and CCS (Fig 9). Cells maintained in FG exhibited increased intracellular granularity at P10 and P30. Significant accumulation of cytosolic vacuoles and granules was observed in cells cultured in CS and ICS, especially after 10 passages.

### Plating efficiency assay of cells cultured in alternative sera

**Fig 10:**
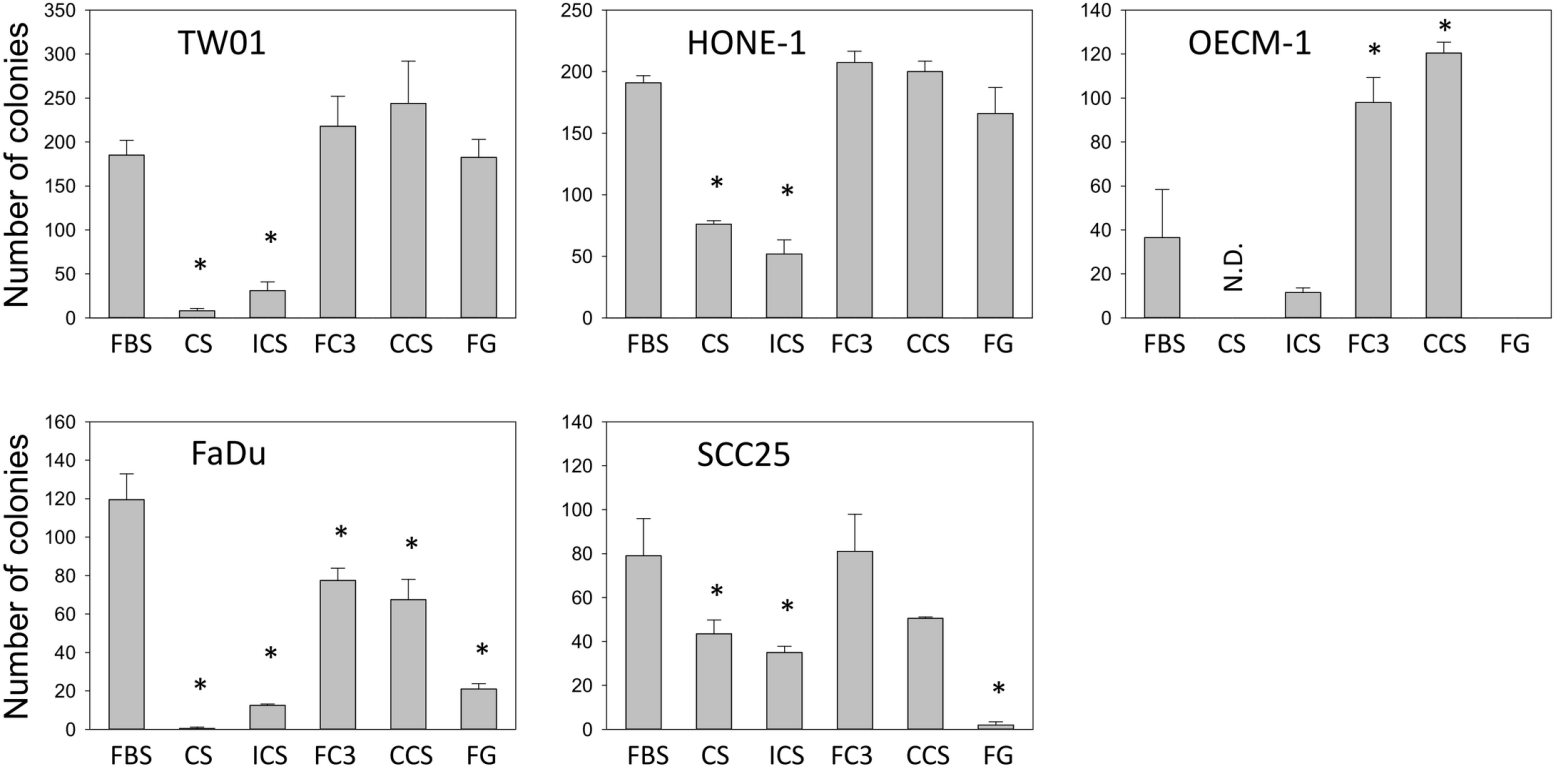
Plating efficiency of cells cultured in alternative sera. Plating efficiency was determined in cells cultured in fetal bovine serum (FBS), calf serum (CS), iron-supplemented calf serum (ICS), FetalClone III (FC3), Cosmic calf serum (CCS), and Fetalgro (FG) serum. Data indicate the average value of duplicates (mean ± SD). *:p<0.05 compared with cells cultured in FBS. N.D.: not determined because subculture terminated at P10 in OECM-1 cells.

The ability to support the growth of cells under very low cell density is a stringent criterion of the quality of culture sera [20]. Many experiments require plating cells at low density so that individual colonies can develop. Therefore, the plating efficiency assay is used by cell culture scientists to test the ability of serum to promote cell growth. The head and neck cell lines cultured in six sera were tested using the plating assay at passage 25. FC3, CCS, and FG exhibited comparable plating efficiency to FBS in TW01 and HONE-1 cells (Fig 10), whereas CS and ICS exhibited significantly low efficiency. For OECM-1 cells, FC3 and CCS exhibited a greater than 2-fold increase in colony formation compared with FBS. OECM-1 maintained in FG exhibited abnormal cell morphology (Fig 6), and no colonies formed in plating assay (Fig 10). Interestingly, although many sera supported the growth of FaDu cells (Fig 3, FaDu), the plating efficiencies of five alternative sera were less than that of FBS. FC3 and CCS exhibited 60% efficiency, whereas other sera exhibited very low efficiencies compared with FBS in FaDu cells. For SCC25 cells, FC3 and CCS had comparable plating efficiencies as FBS, and the efficiencies of other sera were considerably low. This study revealed that one specific serum may have a growth-promoting ability similar to FBS under normal subculture conditions; however, it could have prominent differences from FBS when culturing cells under extremely low-density conditions (compare Figs 3 and 10). In addition, other sera exhibited superior cloning efficiency compared with FBS in specific cells (FC3 and CCS in OECM-1 cells). These results indicate that plating efficiencies in different sera are distinctly cell-type dependent. DOK cells did not form any colonies under such experiment conditions, even in FBS (data not shown).

### Functional characterization of TW01, HONE-1 and FaDu cells cultured in FC3, CCS, and FG

The above results indicate that three FBS alternatives (FC3, CCS, and FG) exhibit comparable performance to FBS in the long-term growth of several cell lines tested in this study (Fig 3). To further characterize the properties of FBS and serum alternatives, cell functional assays, including migration/invasion, anchorage-independent growth, and drug response assays, were analyzed in TW01, HONE-1, and FaDu cells to reveal the characteristics of these sera. These three cells were selected given that their growth rate was similar when cultured in FBS, FC3, CCS and FG (Fig 3). Cells used in the following assays were cultured in medium containing the corresponding sera for greater than 30 passages.

#### Cell migration and invasion assay

**Fig 11:**
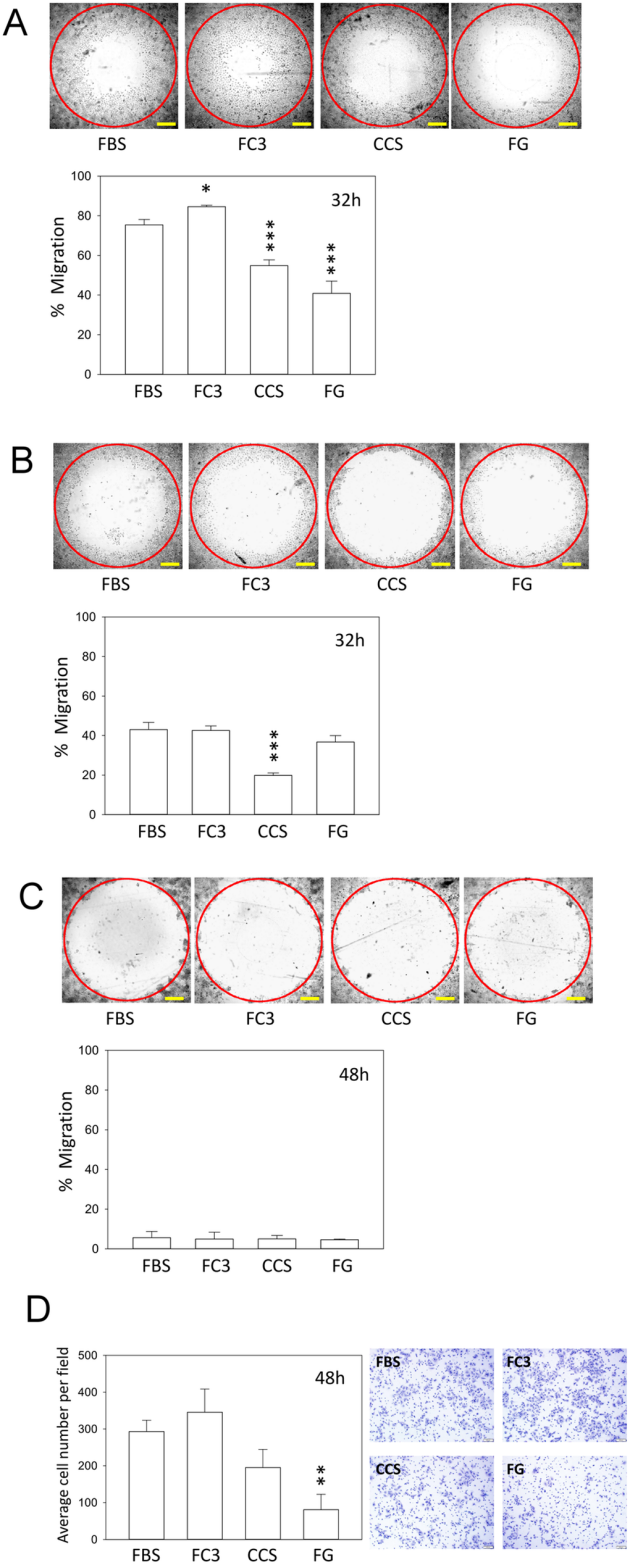
Migration and invasion of cells cultured in alternative sera. Migration of cells cultured in FBS and alternative sera at indicated times. The red circle represents the cell front at t = 0 h when the stopper was removed. Yellow bar represents 200 μm. (A) TW01, (B) HONE-1, and (C) FaDu cells. (D) The invasiveness of TW01 cells was determined by the number of cells that invaded and transmigrated to the lower surface of the transwell membrane at 48 h. Data indicate the average value of three wells (migration) or three culture inserts (invasion) (mean ± SD). *:p<0.05; **:p<0.01; and ***:p<0.001 compared with cells cultured in FBS.

To determine whether serum alternatives affect the migratory capabilities of cells and whether they retain chemotactic effects similar to cells grown in FBS, cell migration and invasion assays were performed. The nasopharyngeal carcinoma cell line TW01 is a highly motile and invasive cell line [23]. TW01 cells cultured in FC3 exhibited increased motility compared with cells grown in FBS (Fig 11A, p = 0.040), whereas cells cultured in CCS and FG exhibited significantly reduced motility compared with cells grown in FBS (p<0.001). The decreased motility of TW01 may in part reflect the fact that their morphology appeared more compact when cultured in CCS and FG (Fig 4). In contrast, HONE-1 cells are not as motile as TW01 cells, and only cells cultured in CCS exhibited reduced motility compared with the other three sera (Fig 11B, p<0.001). FaDu cell migration was very low even after 48 h of incubation (Fig 11C), and no differences were noted among sera. This particular result may also indicate that these FBS substitutes did not alter the migratory property of cells, at least in FaDu, when they intrinsically exhibit low motility. The highly motile TW01 cells were further analyzed using the Matrigel invasion assay to determine whether serum alternatives can attract cells by promoting invasion through the Matrigel matrix (Fig 11D). Cells maintained in FC3 exhibited a slightly increased number of invasive cells, whereas cells cultured in CCS exhibited a reduced number of invasive cells compared with those grown in FBS. However, the results were not statistically significant (compared with FBS, p = 0.435 and p = 0.086, respectively; Fig 11D). Only cells cultured in FG exhibited reduced invasion compared with FBS (p = 0.002). These results indicate that FC3, CCS and FG exhibit chemotactic properties to attract motile cells, albeit to different degrees, compared with FBS. For highly motile cells, such as TW01, the difference between sera could be significant. For cells with low motility, such as FaDu, the difference appears minimal.

#### Anchorage-independent growth assay

**Fig 12:**
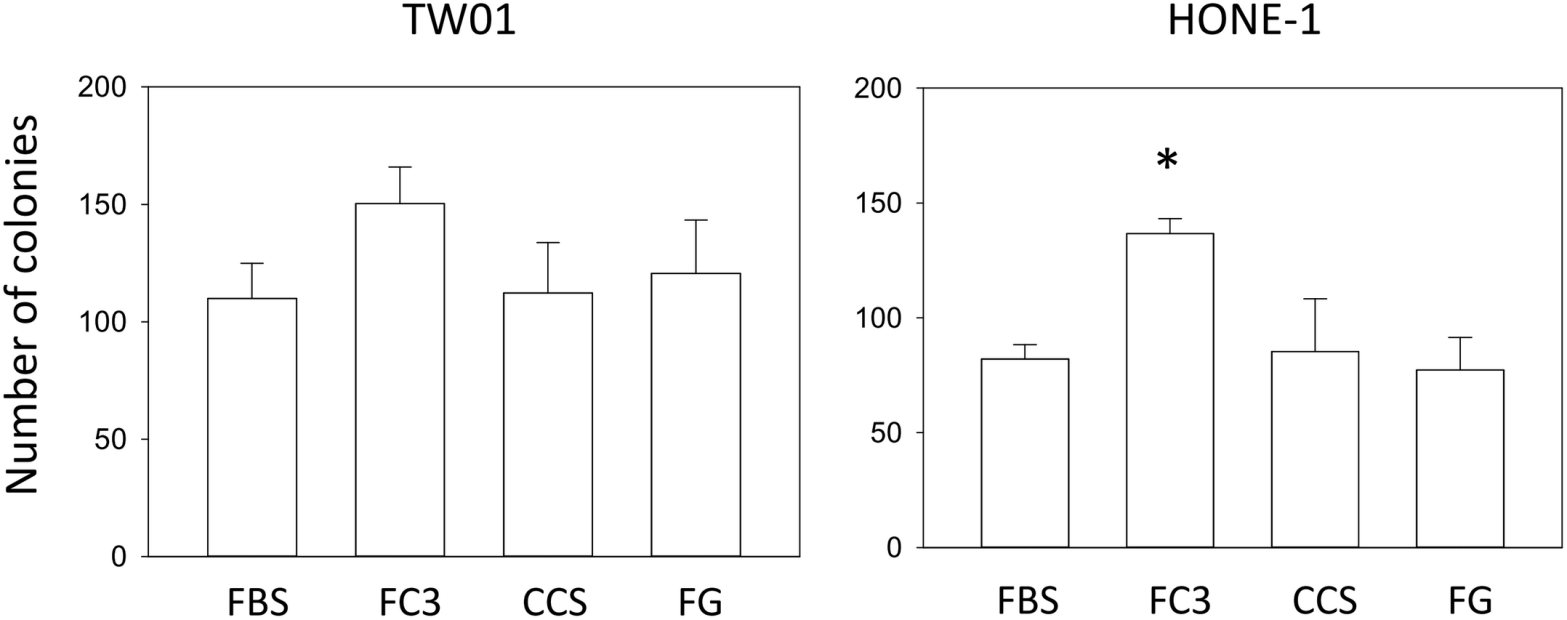
Anchorage-independent growth of cells cultured in alternative sera. Soft-agar colony formation assays were performed in TW01 and HONE-1 cells. Colony numbers were counted after three weeks of growth. Data indicate the average value of triplicates (mean ± SD). *:p = 0.004 compared with cells cultured in FBS.

Anchorage-independent growth is the ability of transformed cells to grow independently of a solid surface. Anchorage-independent growth assays are a well-established, stringent method for characterizing the carcinogenicity of malignant cells *in vitro*. The soft-agar anchorage-independent growth assay was performed to assess differences in performance among sera. The number of TW01 cell colonies formed in FC3, CCS, and FG were comparable to that of FBS, and no significant difference was observed (Fig 12, p>0.05). HONE-1 cells cultured in FC3 exhibit an increased colony number compared with FBS (p = 0.004), whereas cells in CCS and FG exhibited no difference compared with FBS. This result indicates that FC3, CCS and FG offer comparable or better colony-formation ability as FBS in anchorage-independent cell growth. FaDu cells were also tested, but very few colonies were formed (<3 per well) after 3 weeks of growth. Hence, the data were not presented.

#### Cytotoxicity assay

**Fig 13:**
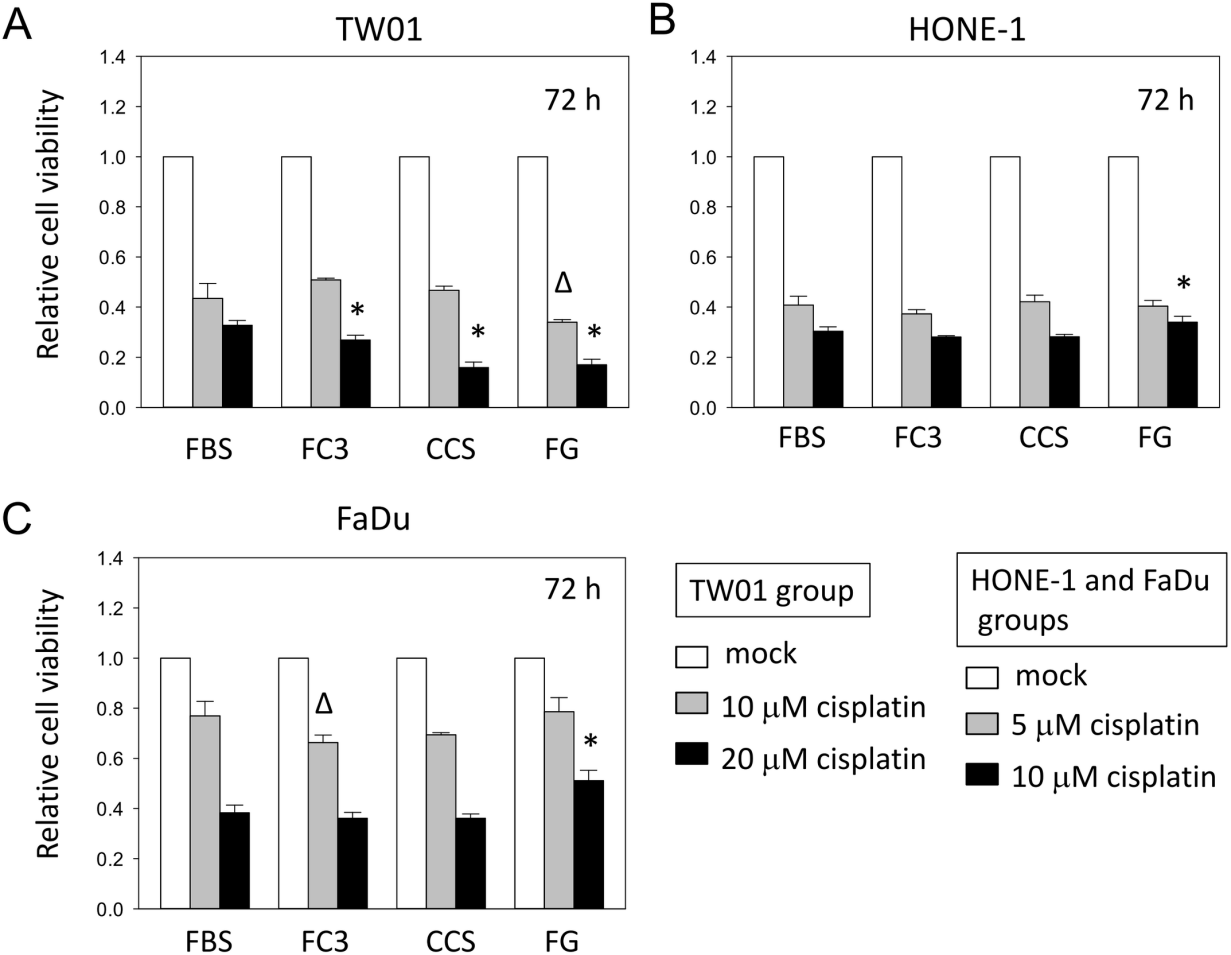
Cytotoxicity assays of cells cultured in alternative sera. Viability assays of cisplatin-treated cells cultured in different sera were performed using a standard MTT assay at 72 h. Cell viability is presented as a relative value when the mock-treated cells were adjusted to 1.0. (A) TW01 cells. Δ: p<0.05 compared with 10 μM cisplatin-treated cells in FBS. *: p<0.05 compared with 20 μM cisplatin-treated cells in FBS. (B) HONE-1 cells. *: p<0.05 compared with 10 μM cisplatin-treated cells in FBS. (C) FaDu cells. Δ: p<0.05 compared with 5 μM cisplatin-treated cells in FBS. *: p<0.05 compared with 10 μM cisplatin-treated cells in FBS. Data indicate the average value of triplicates (mean ± SD).

To determine whether cells responded similarly to drug treatment in alternative sera compared with FBS, we performed a cell cytotoxicity assay with cisplatin treatment in TW01, HONE-1 and FaDu cells (Fig 13). TW01 cells cultured in FC3, CCS, and FG generally responded similarly to cisplatin compared with cells grown in FBS (Fig 13A) but tended to be more sensitive to drug-induced inhibition at high concentration treatments, particularly for cells cultured in FG. The responses to cisplatin treatment were mostly identical in HONE-1 cells cultured in all tested sera. Only an increase in viability was observed in cells cultured in FG with 10 μM cisplatin treatment (Fig 13B). For FaDu cells, the inhibition responses of cisplatin treatment were generally similar, but a slight decrease in the viability of cells cultured in FC3 with 5 μM cisplatin treatment was noted (Fig 13C). Additionally, an increase in FaDu cell viability was noted for FG with 10 μM cisplatin treatment. These results indicate that cells cultured in FC3, CCS, and FG generally have similar responses to cisplatin compared with cells cultured in FBS; however, an enhanced sensitivity to drug inhibition may be observed in some cells.

### Expression profiling of transcription factors in TW01 cells cultured in FBS and FC3

**Fig 14:**
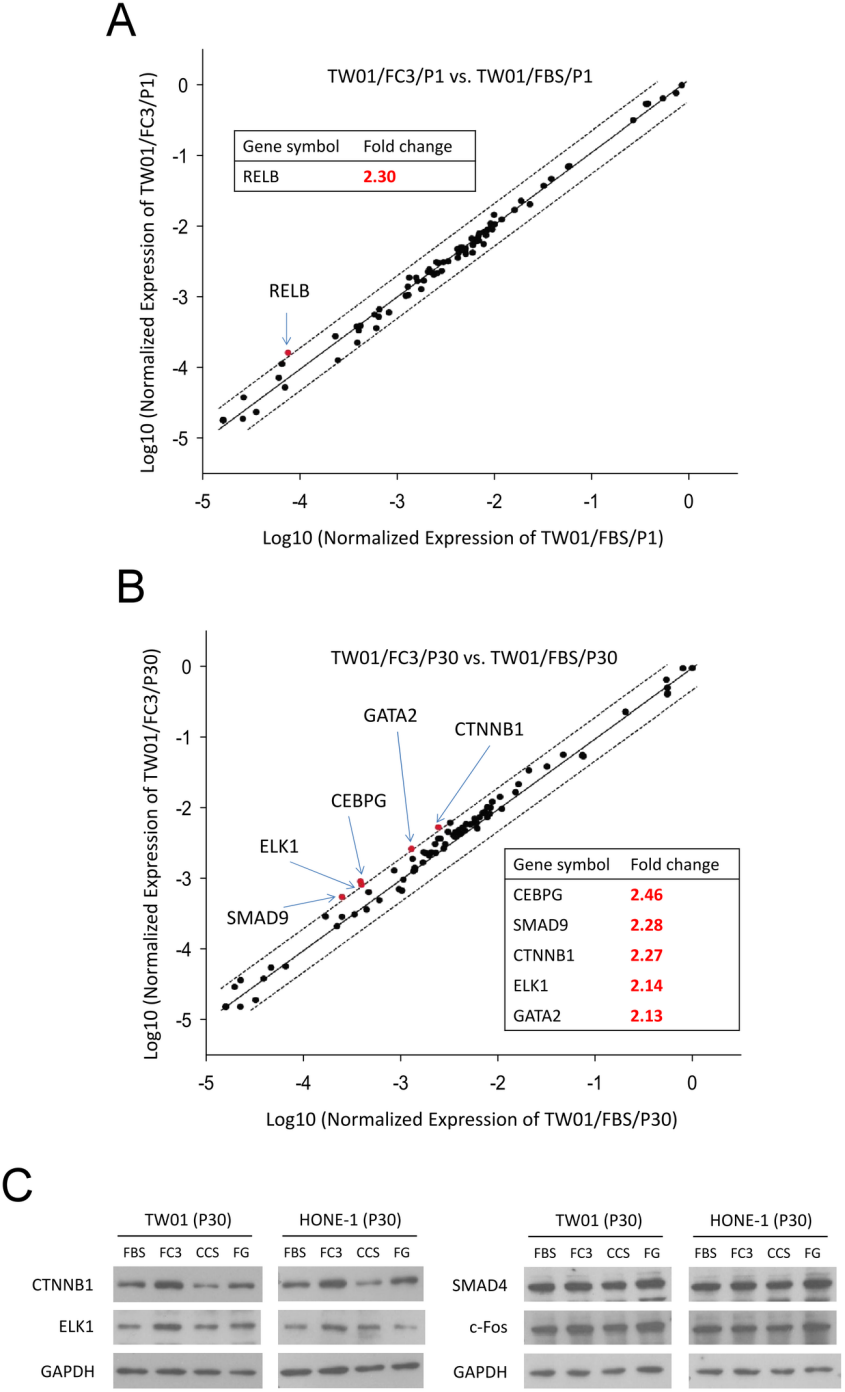
The transcription factor profile of TW01 cells cultured in FBS and FC3. (A) The scatter plots of the transcription factor profile in TW01 P1 cells. (B) The scatter plots of the transcription factor profile in TW01 P30 cells. The central line indicates unchanged gene expression; dotted lines represent the two-fold regulation cut-off. Genes with fold change > 2 indicated. (C) Western blot analysis of CTNNB1, ELK1, SMAD4, and c-Fos expression in TW01 and HONE-1 P30 cells cultured in different sera. GAPDH is detected as a loading control.

In this study, the performance of FC3 was equal to or greater than FBS in many assays. In terms of growth promotion, FC3 appears to be a good serum alternative for replacing FBS in the culture of head and neck cell lines. To further reveal differences in cells cultured in FBS and FC3, a quantitative RT-PCR assay that monitors the expression profile of 84 human transcription factor genes (RT^2^ Profiler PCR Array PAHS-075Z; the complete gene list can be found in: http://www.sabiosciences.com/rt_pcr_product/HTML/PAHS-075Z.html), was performed on TW01 cells at P1 and P30. At P1, the difference in the expression profile between FBS and FC3 was minimal, and only RELB exhibited a greater than 2-fold upregulation in cells cultured in FC3 (Fig 14A). After 30 passages in FBS and FC3, five out of 84 genes (CEBPG, SMAD9, CTNNB1, ELK1, and GATA2) were overexpressed 2.1- to 2.5-fold in cells cultured in FC3 (Fig 14B; for the full unsorted list see S1 File). Except for these genes, the expression profiles of transcription factors between TW01 cells cultured in FC3 and FBS were similar. Western blots assessing CTNNB1 (β-catenin; fold change: 2.27), ELK1 (fold change: 2.14), SMAD4 and c-Fos (fold change less than 1.30) were performed to reveal the expression of these genes in TW01 and HONE-1 cells cultured in FBS, FC3, CCS and FG (Fig 14C). Interestingly, CTNNB1 was upregulated in TW01 and HONE-1 cells cultured in FC3 but downregulated in cells cultured in CCS compared with FBS. ELK1 was also upregulated in TW01 and HONE-1 cells cultured in FC3, confirming quantitative RT-PCR data. SMAD4 and c-Fos expression was relatively constant, and no major variation was noted. These results indicate that the expression profiles of transcription factors of cells cultured in FBS and FC3 were quite similar; however, a few genes were upregulated in TW01 cells cultured in FC3.

## Discussion

Given the ethical concern about the potential suffering of the fetus by the collection practice [2], it had been suggested that researchers should utilize options for cell and tissue culture other than FBS [12]. The efforts to reduce the demands for FBS and the number of bovine fetuses required should be welcomed and supported [28]. The increased demand and limited supply of FBS had caused the price of FBS to increase by greater than 300% in the past few years [6]. In response to the shortage, numerous FBS alternatives have been developed. Sera from other animals (goat, horse, or porcine) have been suggested as potential alternatives to FBS, but their applications were relatively limited given that they can only support the growth of a fraction of cell lines [27, 29–31]. Recently, human serum and human platelet lysates were reported to be a viable FBS substitute in cell culture media [32–34]. The greatest advantage of human serum-derived supplements is that they are non-xenogenic when used with human cell lines. However, potentially due to the limited availability and high prices, their applications are largely confined to the culture of human cells for therapeutic purposes, such as stem cells and mesenchymal stromal cells [35–37]. Numerous serum-free and animal-derived-component-free culture media are also available [38]. However, these media require sophisticated cell adaptation and are not generally suitable for all culture applications. With lower cost and higher availability, calf serum-based FBS alternatives are worthy of investigation as FBS substitutes for most basic studies. The switch from FBS to calf serum-based alternatives might have less impact on cells compared with serum products based on other animals or serum-free products. Additionally, given that part of the calf serum is obtained from donor animals, the ethical issue is mitigated in contrast to FBS. Here, we demonstrated that several calf serum-based alternatives performed comparably to FBS in the culture of head and neck cell lines. These alternative sera may be viable options to replace FBS in the cell culture system.

Newborn calf serum (NBCS) is collected from calves typically 14 days old or younger. NBCS is intrinsically more close to FBS than bovine calf serum, which is collected from calves less than 12 months old [26]. Many serum vendors suggest NBCS as a cost-effective replacement for FBS. However, in this study, NBCS did not provide favorable conditions for replication of head and neck squamous carcinoma cells (Figs 1 and 2). Delayed attachment, restricted extension, and very low proliferation were observed in cells cultured in NBCS. Thus, NBCS performed poorly compared with calf serum (Fig 2). A previous study demonstrated that NBCS is less efficient in the culture of amniotic fluid cells compared with FBS and calf serum [39]. Interestingly, when cells were cultured in medium containing a 1:1 blend of FBS/NBCS, the presence of FBS did not counteract the deleterious effects of NBCS on cells (data not shown). It appears that certain components in the NBCS may have led to the inhibition of cell attachment and extension in NBCS-containing medium, and these factors were not present in either FBS or calf serum. It is not clear why NBCS performed so differently in contrast to FBS and calf serum. The underlying mechanism remains to be elucidated.

Bovine calf serum is readily available and considerably cheaper than FBS. Calf serum is an effective alternative to FBS in the culture of amniotic fluid cells [39]. In this study, the performance of calf serum was significantly reduced compared with FBS. Although it supported the growth of some cells (Fig 3), remarkable morphological changes were noticed (Figs 4–6 and 9). Some very robust cells, such as HSC-3, a human oral squamous carcinoma cell line with high metastatic potential [40], can grow properly, and this cell line can be maintained in medium supplemented with calf serum [40, 41]. In our study, FaDu and SCC25 are the only two cells that did not reveal significant morphological alterations when grown in calf serum, albeit at a lower proliferation rate compared with FBS. It appears that calf serum is not a good choice as an FBS replacement for the cells tested in this study.

Iron-supplemented bovine serum is a viable alternative to FBS in the culture of Chinese hamster ovary cells [42]. Compared with calf serum, iron-supplemented calf serum is a better choice in this study. Iron-supplemented bovine serum exhibited notably better performance than calf serum in TW01 and OECM-1 cells (Fig 3) and had less of an effect on cell morphology compared with calf serum (Fig 6). The performance of iron-supplemented calf serum was constantly less than that of FBS. For robust cell lines, such as FaDu and SCC25, iron-supplemented calf serum may be a suitable FBS replacement when vigorous cell growth is not required.

The performance of Fetalgro was similar or equal to that of FBS in five cell lines tested in this study (Fig 3). The only exception is OECM-1 cells, in which a dramatic morphology change was observed when cultured in Fetalgro-containing medium (Fig 6). Fetalgro supported the long-term replication of cells in this study. Fetalgro exhibited comparable colony formation ability as FBS in TW01 and HONE-1 cells in the anchorage-independent growth assay (Fig 12) and performed similar to FBS in the cytotoxicity assay (Fig 13). The list price of Fetalgro is among the range of newborn calf serum from other major serum vendors. Therefore, Fetalgro is an economically appealing choice as an FBS replacement for culturing suitable cell lines.

Cosmic calf serum exhibited growth-promoting capabilities generally equal to FBS in five tested cell lines and outperformed FBS in FaDu cells in this study (Fig 3). No major morphological changes were observed in OECM-1, FaDu, SCC255, and DOK cells cultured in Cosmic calf serum (Figs 6–9). However, the colonies of TW01 and HONE-1 nasopharyngeal carcinoma cells were more compact compared with cells grown in FBS (Figs 4 and 5). The plating efficiency assay also revealed that Cosmic calf serum is as efficient as FBS for promoting cell growth of the three cell lines under stringent conditions (Fig 10). Cosmic calf serum exhibited comparable colony formation to that of FBS in the anchorage-independent growth assay (Fig 12) and performed similar to FBS in the cytotoxicity assay (Fig 13). Cosmic calf serum also induced migration and invasion of motile cells, but to a lesser degree compared with FBS (Fig 11). In general, Cosmic calf serum is a good choice for replacing FBS in the culture of head and neck cell lines.

FetalClone III exhibited very good performance in this study. FetalClone III had growth-promoting abilities equal to FBS in TW01, HONE-1, SCC25, and DOK cells (Fig 3) and outperformed FBS in OECM-1 and FaDu cells. In OECM-1 cells, FetalClone III resulted in a 50% increase in cell proliferation compared with FBS. No discernible morphological changes were noticed in all tested cells cultured in FetalClone III (Figs 4–9). FetalClone III also exhibited comparable plating efficiency as FBS in three of the tested cells (Fig 10) and outperformed FBS in OECM-1 cells. FetalClone III exhibited comparable colony formation ability as FBS in the anchorage-independent growth assay of TW01 cells and outperformed FBS in HONE-1 cells (Fig 12). FetalClone III also performed similar to FBS in the cisplatin-induced cytotoxicity assay (Fig 13). In migration and invasion assays, FetalClone III exhibited an increased chemotactic effect on TW01 cells compared with FBS (Fig 11). Although FetalClone III is a bovine calf serum-based product, it is different from others in that it is processed to remove the immunoglobulin content. Thus, FetalClone serum has antibody concentration as low as FBS [43]. This feature is an advantage in culture applications where the interference of bovine immunoglobulin is a concern. Based on the results of this study, FetalClone III is a good FBS alternative for culturing head and neck cell lines.

In addition to the head and neck cell lines tested in this study, some of these alternative sera support the growth of several types of cells, including fibroblasts, epithelial cells, and hybridomas [3, 13]. Their applications in culture of different cell types could be much wider than the observations made in this study. On the other hand, our study also indicated that even cells from similar origins (epithelium of head and neck) could have very different responses to a specific alternative serum. Therefore, caution should be taken in deciding which FBS alternative to use in the culture of specific cell types. This study revealed that although some sera seemed to promote the growth of cells, significant morphological changes were observed in these cells, indicating that their physiological characteristics may be different from cells in FBS (Figs 4–9). Discrepancies in chemotactic response were observed in cells cultured in different sera despite a comparable growth rate in these sera (Figs 3 and 11). A shift in the expression profile was also noted between TW01 cells cultured in FBS and FC3 (Fig 14). Therefore, the suitability of an FBS alternative for a specific cell line should be determined by each investigator.

In summary, our study indicates that several bovine calf serum-based FBS alternatives exhibited growth promoting capabilities comparable or superior to that of FBS in the culture of head and neck squamous cell lines. Most importantly, these sera can support the long-term growth of cells and exhibit plating efficiencies comparable to that of FBS. The results of functional assays also indicated that cells cultured in FBS alternatives exhibited anchorage-independent growth and drug sensitivities similar to FBS. These alternatives are more readily available and considerably less costly than FBS; a substantial reduction of the cost can be realized. A previous report demonstrated that the growth-promoting performance of FBS alternatives was more consistent from lot to lot than FBS [3]. Although the ultimate goal of cell and tissue culture should be the complete elimination of animal-derived components in the culture system, there is still a long way to go before most cells could be grown synthetic serum-free media. Until then, these bovine calf serum-based alternatives may provide a good option for replacing FBS in the cell culture system.

## Supporting information

S1 File The expression lists of 84 transcription factor genes of TW01 cells cultured in FBS and FC3 at passage 1 and 30. The expression profiles of 84 transcription factors in TW01 cells were detected by RT^2^-PCR arrays.
(ZIP)

## References

[1] Bettger WJ, McKeehan WL. Mechanisms of cellular nutrition. Physiol Rev. 1986;66:1–35. . 3511479 10.1152/physrev.1986.66.1.1

[2] van der Valk J, Mellor D, Brands R, Fischer R, Gruber F, Gstraunthaler G, et al The humane collection of fetal bovine serum and possibilities for serum-free cell and tissue culture. Toxicol In Vitro. 2004;18:1–12. . 14630056 10.1016/j.tiv.2003.08.009

[3] Hyclone. Growth comparison studies between FBS and other serum products. Thermo Scientific. 2010 [cited 2016 Nov 28]. Available from: http://apps.thermoscientific.com/media/BID/BPP/appnotes/serumgrowthcomparison/growth-comparison-fbs-vs-other-serum.pdf.

[4] Jayme DW, Blackman KE. Culture media for propagation of mammalian cells, viruses, and other biologicals. Adv Biotechnol Process. 1985;5:1–30. . 2417609

[5] Jayme DW, Epstein DA, Conrad DR. Fetal bovine serum alternatives. Nature. 1988;334:547–8. doi: 10.1038/334547a0 . 3405300 10.1038/334547a0

[6] RMBIO. Fetal bovine serum: supply and demand for US FBS. Rocky Mountain Biologicals. 2016 [cited 2016 Nov. 24]. Available from: https://www.rmbio.com/fetal-bovine-serum-supply-and-demand-for-us-fbs.

[7] Jochems CEA, van der Valk JBF, Stafleu FR, Baumans V. The use of fetal bovine serum: Ethical or scientific problem? Altern Lab Anim. 2002;30:219–27. . 11971757 10.1177/026119290203000208

[8] Johnston S, Siegel C. Comparison of a serum replacement (Omni Serum) and fetal bovine serum in cell cultures used to isolate herpes simplex virus from clinical specimens. J Clin Microbiol. 1990;28:643–5. ; PubMed Central PMCID: PMCPMC267768. 2159017 10.1128/jcm.28.4.643-645.1990 PMC267768

[9] Dahling DR, Wright BA. Comparison of fortified calf serum, serum substitutes and fetal calf serum with or without extenders for propagation of cell cultures for virus plaque assays. J Virol Methods. 1990;27:287–94. . 2324238 10.1016/0166-0934(90)90097-y

[10] Hodgson J. To treat or not to treat—That is the question for serum. Nat Biotechnol. 1995;13:333 doi: 10.1038/nbt0495-333 . 10.1038/nbt0495-333 9634775

[11] Nielsen O. Changing sera mind-set. Nat Biotechnol. 1995;13:626 doi: 10.1038/nbt0795-626 .

[12] Gstraunthaler G, Lindl T, van der Valk J. A plea to reduce or replace fetal bovine serum in cell culture media. Cytotechnology. 2013;65:791–3. doi: 10.1007/s10616-013-9633-8 ; PubMed Central PMCID: PMCPMC3967615. 23975256 10.1007/s10616-013-9633-8 PMC3967615

[13] RMBIO. Fetalgro—bovine growth serum. Rocky Mountain Biologicals. 2016 [cited 2016 Dec 07]. Available from: https://www.rmbio.com/document/fetalgro%20flier.pdf.

[14] Lin CT, Wong CI, Chan WY, Tzung KW, Ho JK, Hsu MM, et al Establishment and characterization of two nasopharyngeal carcinoma cell lines. Lab Invest. 1990;62:713–24. . 2162997

[15] Glaser R, Zhang HY, Yao KT, Zhu HC, Wang FX, Li GY, et al Two epithelial tumor cell lines (HNE-1 and HONE-1) latently infected with Epstein-Barr virus that were derived from nasopharyngeal carcinomas. Proc Natl Acad Sci USA. 1989;86:9524–8. . 2556716 10.1073/pnas.86.23.9524 PMC298529

[16] Yang CY, Meng CL. Regulation of PG synthase by EGF and PDGF in human oral, breast, stomach, and fibrosarcoma cancer cell lines. J Dent Res. 1994;73:1407–15. doi: 10.1177/00220345940730080301 8083436 10.1177/00220345940730080301

[17] Rangan SR. A new human cell line (FaDu) from a hypopharyngeal carcinoma. Cancer. 1972;29:117–21. . 4332311 10.1002/1097-0142(197201)29:1<117::aid-cncr2820290119>3.0.co;2-r

[18] Rheinwald JG, Beckett MA. Tumorigenic keratinocyte lines requiring anchorage and fibroblast support cultured from human squamous cell carcinomas. Cancer Res. 1981;41:1657–63. . 7214336

[19] Chang SE, Foster S, Betts D, Marnock WE. DOK, a cell line established from human dysplastic oral mucosa, shows a partially transformed non-malignant phenotype. Int J Cancer. 1992;52:896–902. Epub 1992/12/02. . 1459732 10.1002/ijc.2910520612

[20] Goodheart CR, Castro BC, Giviers A, Regnier PR. Plating efficiency for primary hamster embryo cells as an index of efficacy of fetal bovne serum for cell culture. Appl Microbiol. 1973;26:525–8. ; PubMed Central PMCID: PMCPMC379841. 4584591 10.1128/am.26.4.525-528.1973 PMC379841

[21] Fang CY, Wu CC, Hsu HY, Chuang HY, Huang SY, Tsai CH, et al EGCG inhibits proliferation, invasiveness and tumor growth by up-regulation of adhesion molecules, suppression of gelatinases activity, and induction of apoptosis in nasopharyngeal carcinoma cells. Int J Mol Sci. 2015;16:2530–58. doi: 10.3390/ijms16022530 ; PubMed Central PMCID: PMC4346850. 25625511 10.3390/ijms16022530 PMC4346850

[22] Yue PY, Leung EP, Mak NK, Wong RN. A simplified method for quantifying cell migration/wound healing in 96-well plates. J Biomol Screen. 2010;15:427–33. doi: 10.1177/1087057110361772 . 20208035 10.1177/1087057110361772

[23] Fang CY, Lee CH, Wu CC, Chang YT, Yu SL, Chou SP, et al Recurrent chemical reactivations of EBV promotes genome instability and enhances tumor progression of nasopharyngeal carcinoma cells. Int J Cancer. 2009;124:2016–25. Epub 2009/01/10. doi: 10.1002/ijc.24179 . 19132751 10.1002/ijc.24179

[24] Kakuguchi W, Kitamura T, Kuroshima T, Ishikawa M, Kitagawa Y, Totsuka Y, et al HuR knockdown changes the oncogenic potential of oral cancer cells. Mol Cancer Res. 2010;8:520–8. doi: 10.1158/1541-7786.MCR-09-0367 . 20332213 10.1158/1541-7786.MCR-09-0367

[25] Fang CY, Huang SY, Wu CC, Hsu HY, Chou SP, Tsai CH, et al The synergistic effect of chemical carcinogens enhances Epstein-Barr virus reactivation and tumor progression of nasopharyngeal carcinoma cells. PLoS One. 2012;7:e44810 Epub 2012/10/02. doi: 10.1371/journal.pone.0044810 . 23024765 10.1371/journal.pone.0044810 PMC3443098

[26] RMBIO. Comparison of FBS and other bovine serums. Rocky Mountain Biologicals. 2016 [cited 2016 Dec 07]. Available from: https://www.rmbio.com/comparison-of-fbs-and-other-bovine-serums.

[27] Zamansky GB, Arundel C, Nagasawa H, Little JB. Adaptation of human diploid fibroblasts in vitro to serum from different sources. J Cell Sci. 1983;61:289–97. . 6885940 10.1242/jcs.61.1.289

[28] Gstraunthaler G. Alternatives to the use of fetal bovine serum: serum-free cell culture. ALTEX. 2003;20:275–81. . 14671707

[29] Paranjape S. Goat serum: an alternative to fetal bovine serum in biomedical research. Indian J Exp Biol. 2004;42:26–35. . 15274477

[30] Franke J, Abs V, Zizzadoro C, Abraham G. Comparative study of the effects of fetal bovine serum versus horse serum on growth and differentiation of primary equine bronchial fibroblasts. BMC Vet Res. 2014;10:119 doi: 10.1186/1746-6148-10-119 ; PubMed Central PMCID: PMCPMC4040117. 24886635 10.1186/1746-6148-10-119 PMC4040117

[31] Ziegler A, Everett H, Hamza E, Garbani M, Gerber V, Marti E, et al Equine dendritic cells generated with horse serum have enhanced functionality in comparison to dendritic cells generated with fetal bovine serum. BMC Vet Res. 2016;12(1):254 doi: 10.1186/s12917-016-0880-8 ; PubMed Central PMCID: PMCPMC5111218. 27846835 10.1186/s12917-016-0880-8 PMC5111218

[32] Canovas D, Bird N. Human AB serum as an alternative to fetal bovine serum for endothelial and cancer cell culture. ALTEX. 2012;29:426–8. . 23138512 10.14573/altex.2012.4.426

[33] Rauch C, Feifel E, Amann EM, Spotl HP, Schennach H, Pfaller W, et al Alternatives to the use of fetal bovine serum: human platelet lysates as a serum substitute in cell culture media. ALTEX. 2011;28:305–16. . 22130485 10.14573/altex.2011.4.305

[34] Witzeneder K, Lindenmair A, Gabriel C, Holler K, Theiss D, Redl H, et al Human-derived alternatives to fetal bovine serum in cell culture. Transfus Med Hemoth. 2013;40:417–23. doi: 10.1159/000356236 . 10.1159/000356236 PMC3901599 24474892

[35] Bieback K. Platelet lysate as replacement for fetal bovine serum in mesenchymal stromal cell cultures. Transfus Med Hemoth. 2013;40(5):326–35. doi: 10.1159/000354061 . 10.1159/000354061 PMC3822281 24273486

[36] Hemeda H, Giebel B, Wagner W. Evaluation of human platelet lysate versus fetal bovine serum for culture of mesenchymal stromal cells. Cytotherapy. 2014;16:170–80. doi: 10.1016/j.jcyt.2013.11.004 . 24438898 10.1016/j.jcyt.2013.11.004

[37] Shanskii YD, Sergeeva NS, Sviridova IK, Kirakozov MS, Kirsanova VA, Akhmedova SA, et al Human platelet lysate as a promising growth-stimulating additive for culturing of stem cells and other cell types. Bull Exp Biol Med. 2013;156:146–51. doi: 10.1007/s10517-013-2298-7 . 24319712 10.1007/s10517-013-2298-7

[38] Brunner D, Frank J, Appl H, Schoffl H, Pfaller W, Gstraunthaler G. Serum-free cell culture: the serum-free media interactive online database. ALTEX. 2010;27:53–62. . 20390239 10.14573/altex.2010.1.53

[39] Heaton DE, Snape BM, Fennell SJ, Marsden HB. Aseptically collected calf serum as an effective alternative to fetal calf serum in the culture of amniotic fluid cells. Prenat Diagn. 1982;2:281–8. . 7156024 10.1002/pd.1970020407

[40] Momose F, Araida T, Negishi A, Ichijo H, Shioda S, Sasaki S. Variant sublines with different metastatic potentials selected in nude mice from human oral squamous cell carcinomas. J Oral Pathol Med. 1989;18:391–5. . 2585303 10.1111/j.1600-0714.1989.tb01570.x

[41] JCRB0623 HSC-3. JCRB Cell Bank. [cited 2016 Dec 09]. Available from: http://cellbank.nibiohn.go.jp/~cellbank/en/search_res_det.cgi?ID=468.

[42] Oberly TJ, Rexroat MA, Richardson KK. Iron-supplemented bovine serum as an alternative to fetal bovine serum in the CHO/HGPRT mutation assay. Mutat Res. 1990;244:105–9. . 2355932 10.1016/0165-7992(90)90057-q

[43] Hyclone. Thermo Scientific HyClone FetalClone I, II and III: serum alternatives to FBS. Thermo Scientific. 2013 [cited 2016 Dec 10]. Available from: https://static.thermoscientific.com/images/D22604~.pdf.

